# Development and psychometric validation of measures to assess the impact of phenylketonuria and its dietary treatment on patients’ and parents’ quality of life: the phenylketonuria – quality of life (PKU-QOL) questionnaires

**DOI:** 10.1186/s13023-015-0261-6

**Published:** 2015-05-10

**Authors:** Antoine Regnault, Alberto Burlina, Amy Cunningham, Esther Bettiol, Flavie Moreau-Stucker, Khadra Benmedjahed, Annet M Bosch

**Affiliations:** Mapi, Health Economics & Outcomes Research and Strategic Market Access, 27 rue de la Villette, Lyon, France; Division of Metabolic Diseases, Department of Paediatrics, University Hospital of Padova, Padova, Italy; Hayward Genetics Center, Tulane University School of Medicine, New Orleans, Louisiana USA; Infection Control Program, University of Geneva Hospitals and Faculty of Medicine, Geneva, Switzerland; EMD Serono Inc, Billerica, Massachusetts USA; Department of Pediatrics, Division of Metabolic Disorders, Academic Medical Centre, University of Amsterdam, Amsterdam, The Netherlands

**Keywords:** Rare disease, Phenylketonuria, Questionnaires, Health-related quality of life, Children, Adolescents, Parents, Cross-cultural adaptation, Simultaneous development, Psychometric validation

## Abstract

**Background:**

The aim of our study was to develop and validate the first set of PKU-specific Health-related Quality of Life (HRQoL) questionnaires that: 1) were developed for patients with PKU and their parents, 2) cover the physical, emotional, and social impacts of PKU and its treatment on patients’ lives, 3) are age specific (Child PKU-QOL, Adolescent PKU-QOL, Adult PKU-QOL), 4) enable the evaluation of the HRQoL of children by their parents (Parent PKU-QOL), and 5) have been cross-culturally adapted for use in seven countries (i.e. France, Germany, Italy, The Netherlands, Spain, Turkey and the UK).

**Methods:**

The PKU-QOL questionnaires were developed according to reference methods including patients’, parents’ and healthcare professionals’ interviews; testing in a pilot study (qualitative step in six countries), and linguistic validation of the finalised pilot versions in Turkish. For finalisation and psychometric validation, the pilot versions were included in a multicentre, prospective, non-interventional, observational study conducted in 34 sites in France, Germany, Italy, The Netherlands, Spain, Turkey and the UK. Iterative multi-trait analyses were conducted. Psychometric properties were assessed (concurrent and clinical validity, internal consistency reliability and test-retest reliability).

**Results:**

Data from 559 subjects (306 patients, 253 parents) were analysed. After finalisation, the PKU-QOL questionnaires included 40 items (Child PKU-QOL), 58 items (Adolescent PKU-QOL), 65 items (Adult PKU-QOL) and 54 items (Parent PKU-QOL), distributed in four modules: PKU symptoms, PKU in general, administration of Phe-free protein supplements and dietary protein restriction. The measurement properties of the Adolescent, Adult and Parent PKU-QOL questionnaires were overall fairly satisfactory, but weaker for the Child questionnaire.

**Conclusions:**

The four PKU-QOL questionnaires developed for different ages (Child PKU-QOL, Adolescent PKU-QOL, Adult PKU-QOL), and for parents of children with PKU (Parent PKU-QOL) are valid and reliable instruments for assessing the multifaceted impact of PKU on patients of different age groups (children, adolescents and adults) and their parents, and are available for use in seven countries. They are very promising tools to explore how patients’ perceptions evolve with age, to increase knowledge of the impact of PKU on patients and parents in different countries, and to help monitor the effect of therapeutic strategies.

**Electronic supplementary material:**

The online version of this article (doi:10.1186/s13023-015-0261-6) contains supplementary material, which is available to authorized users.

## Background

Phenylketonuria (PKU, OMIM 261600) is a rare genetic disorder (with an incidence of 1 in 10,000 births in Europe [1]) characterised by a deficiency of the hepatic enzyme, phenylalanine hydroxylase (PAH, EC 1.14.16.1), responsible for the conversion of the essential amino acid phenylalanine (Phe) into tyrosine. The absence of or deficiency in PAH results in increased blood concentrations of Phe and toxic accumulation in the brain. If left untreated, PKU may lead to intellectual impairment, deficit in cognitive functions, seizures, behavioural problems and psychiatric symptoms [2,3]. Historically, PKU can be classified by Phe level at the time of diagnosis, with levels 360–1200 μmol/L being classified as mild-moderate PKU and >1200 μmol/L as classical PKU.

With the implementation of new-born screening programmes and early diagnosis and treatment, patients with PKU can develop normally. Current treatment for PKU includes a life-long diet highly restrictive in Phe, supported nutritionally with a Phe-free protein supplement (medical food, metabolic formula, amino acid mixtures), and excluding high protein foods such as meat, fish, eggs, cheese, milk products and bread [4]. Recent and on-going development of pharmacological treatment tools may allow modification of dietary restriction, but do not yet consistently allow discontinuation of traditional diet therapy.

However, even when treated, PKU may have an impact on neurocognitive and psychosocial outcomes. In a meta-analysis examining neuropsychological outcomes in early and continuously treated adolescents and adults, Moyle et al. [5] demonstrated that patients with PKU differed significantly from controls on Full-Scale IQ, processing speed, attention, inhibition, and motor control. Psychological disorders such as low self-esteem, lower achievement motivation, decreased autonomy and decreased social competence have been reported in early-treated children, and adolescents and adults may be at risk for depressed mood, generalized anxiety, and social isolation [6,7].

The management of PKU is complex, requiring adherence to diet therapy and Phe-free protein supplement intake, regular collection of blood samples, recording of food intake, and regular visits to the PKU clinic [8]. Adherence to the diet is especially important during the early childhood years since cognitive outcomes are closely related to the control of blood phenylalanine levels [9], and should be maintained through adulthood to protect from neuropsychological dysfunction [10,11,5,12]. However, the strict low-Phe diet imposes a burden on patients and their families and has been associated with dietary non-compliance, especially in adolescents and young adults [10,13-15]. Primary obstacles to better adherence include time constraints and stress associated with food preparation and record-keeping, and the restrictions imposed on social life [13].

For years, preventing intellectual impairment has been the primary goal of PKU treatment. At present, ‘a life as normal as possible’ is an additional goal of therapy [16], aiming not only for normal neuropsychological test outcomes, but also for normal quality of life [12]. Health-related quality of life (HRQoL) has been defined as a broad and multidimensional concept representing the patient’s subjective perception of the impact of his disease and its treatment(s) on his daily life, physical, psychological and social functioning and well-being [17]. HRQoL studies focusing on patients with PKU and their parents are still scarce [18-22,11,23-27]. Most studies suggest that the HRQoL of patients with PKU is comparable to that of the general population [18,19,21,11,23,24,26] with the exception of a lower HRQoL demonstrated in a group of Italian children [20], a low score on the cognitive domain in adults [28], and of severe to moderate distress in 45% of non-compliant adults patients [18]. Parents of children with PKU perceive their HRQoL positively overall even though it may be affected by the emotional and social impact of parenting a child with PKU [22,25]. These studies all used generic measures of HRQoL, i.e. questionnaires intended for use irrespective of the underlying disease. The positive HRQoL results observed in patients with PKU may, at least in part, be caused by the fact that these questionnaires may be not be sensitive enough to allow detection of the specific or subtle problems of patients with PKU[29-31]. A PKU-specific HRQoL questionnaire developed with and for patients with PKU will allow their experience to be more accurately captured, with all its complexity. Therefore, such an instrument will be able to detect decrements in specific domains of the life of patients with PKU as well as potential improvements in these domains due to therapeutic interventions. In addition, a PKU-specific questionnaire will make more sense to patients with PKU, which will certainly lead them to naturally adhere to the questionnaire and therefore generate better data.

The aim of our study was to develop and validate the first set of PKU-specific HRQoL questionnaires that: 1) were developed with patients with PKU and their caregivers for children, 2) identify the physical, emotional, and social impacts characteristic for PKU and its treatment on patients’ lives, 3) are age specific (Child PKU-QOL, Adolescent PKU-QOL, Adult PKU-QOL), 4) enable the evaluation of the HRQoL of children by their parents (Parent PKU-QOL), and 5) are cross-culturally adapted to seven countries (i.e. France, Germany, Italy, The Netherlands, Spain, Turkey and the UK).

## Methods

### Development of the questionnaires

**Figure 1:**
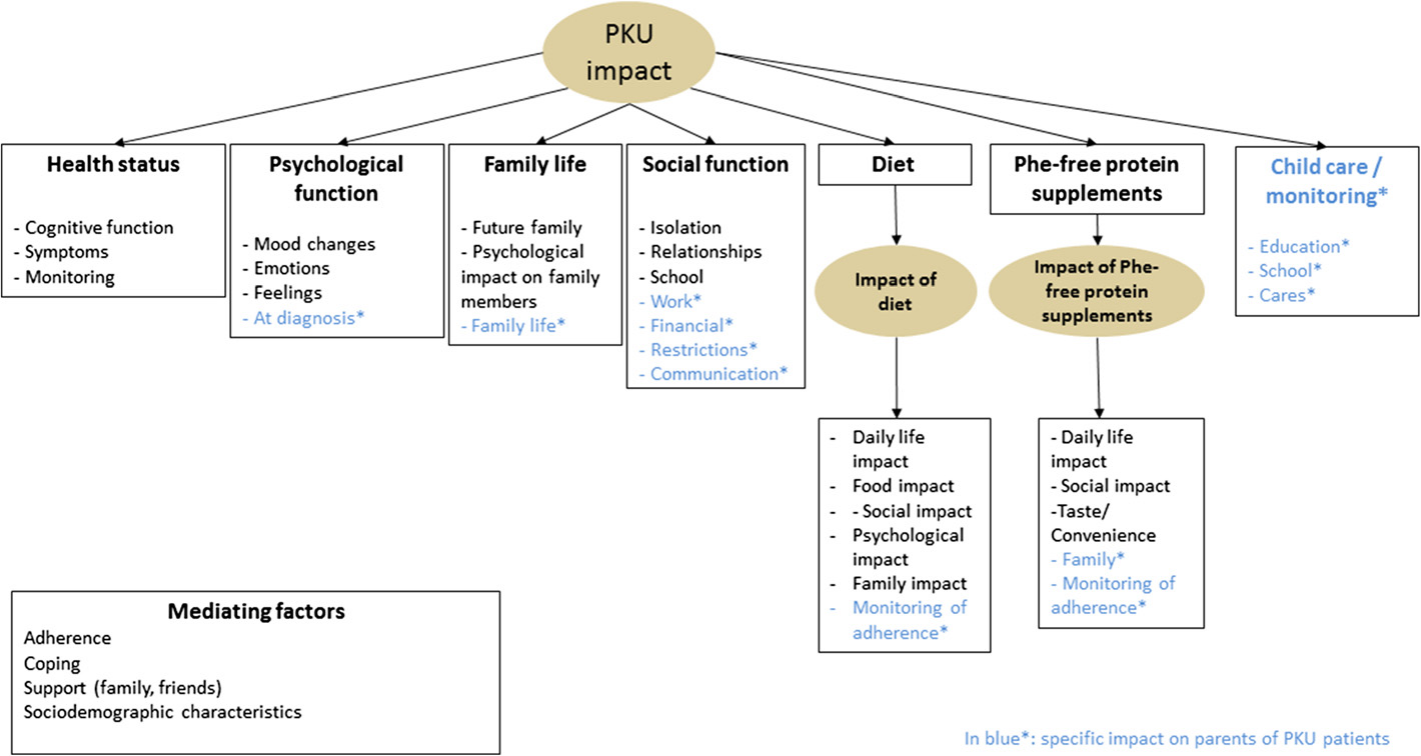
Conceptual model of the impact of phenylketonuria (PKU) and its treatment on patients and their parents. From the patients’ perspective, PKU can have an impact on health status, psychological function, family life and social function. PKU treatment was also reported as having either a negative impact or no impact (patients who indicated that they did not know any other way of living and had coped with their disease).

The questionnaires were developed in a sequential four step approach. The first step included exploratory interviews (in 2007) with patients with PKU, parents (and healthcare professionals). Interviews were carried out simultaneously in France, Germany, Spain and the UK (Table 1). These interviews collected disease-related concepts important to patients and their parents regarding the impact of PKU and its treatment (i.e. diet and Phe-free protein supplements) on their life. The thematic analysis of the qualitative information collected with both patients, parents and healthcare professionals during this first phase allowed the creation of conceptual models of the impact of PKU and its treatment for patients and parents (Figure 1).

**Table 1:** **Development and validation phases of the PKU-QOL questionnaire – disposition of subjects by phase and country**

	**France**	**Germany**	**Italy**	**Netherlands**	**Spain**	**Turkey**	**UK**	**Total**
**Exploratory interviews**								
Nutritionists	**4**	**3**	**-**	**-**	**4**	**-**	**6**	**17**
Pediatricians	**3**	**4**	**-**	**-**	**3**	**-**	**3**	**13**
Adolescents 13–17 years with PKU	**4**	**4**	**-**	**-**	**4**	**-**	**4**	**16**
Adults with PKU	**4**	**4**	**-**	**-**	**4**	**-**	**4**	**16**
Parents of children 4–12 years with PKU	**4**	**4**	**-**	**-**	**4**	**-**	**4**	**16**
Parents of adolescents 13–17 years with PKU	**0**	**0**	**-**	**-**	**3**	**-**	**0**	**3**
**Comprehension testing**								
Children 6–11 years with PKU	**3**	**3**	**3**	**3**	**3**	**-**	**3**	**18**
Adolescents 12–17 years with PKU	**4**	**4**	**4**	**4**	**4**	**-**	**4**	**24**
Adults with PKU	**5**	**4**	**4**	**3**	**4**	**-**	**4**	**24**
Parents of children with PKU	**5**	**5**	**5**	**5**	**5**	**-**	**6**	**31**
**Linguistic validation**								
Children 6–11 years with PKU	**-**	**-**	**-**	**-**	**-**	**3**	**-**	**3**
Adolescent 12–17 years with PKU	**-**	**-**	**-**	**-**	**-**	**3**	**-**	**3**
Adults with PKU	**-**	**-**	**-**	**-**	**-**	**3**	**-**	**3**
Parents of children with PKU	**-**	**-**	**-**	**-**	**-**	**3**	**-**	**3**
**Psychometric Validation study**								
Children 9–11 year with PKU	**13**	**19**	**15**	**7**	**20**	**14**	**4**	**92**
Adolescents 12–17 years with PKU	**10**	**20**	**26**	**10**	**20**	**20**	**4**	**110**
Adults with PKU	**18**	**21**	**22**	**7**	**21**	**8**	**7**	**104**
Parents of children ≤8 years with PKU	**10**	**8**	**8**	**6**	**8**	**6**	**6**	**52**
Parents of children 9–11 years with PKU	**13**	**19**	**15**	**7**	**20**	**4**	**14**	**92**
Parents of adolescents with PKU	**10**	**19**	**26**	**10**	**20**	**4**	**20**	**109**

As a second step, question items were generated based on the conceptual models in six languages (Dutch [The Netherlands], English [UK], French [France], German [Germany], Italian [Italy] and Spanish [Spain]). These formed the content of four questionnaires: one for children, adolescents, adults and parents (to assess their child’s as well as their own HRQoL). After item generation, the preliminary test versions of the questionnaires were reviewed and refined by native speakers of each language to produce the final test versions. The third step, comprehension testing, aimed at checking the acceptability of the questionnaires, the relevance and understanding of the items and response choices, the comprehensiveness of the questions, and opinions regarding format and layout, and suggestions for rewording of questionnaires if applicable. Ninety-seven interviews were conducted in The Netherlands, Germany, Italy, Spain, France and the UK (Table 1). Overall, the questionnaires were well accepted and understood by participants. However, children younger than 9 years of age showed some difficulty in understanding the questionnaire by themselves, and therefore the age range of the child version, originally planned for children aged 6–11 years, was narrowed to 9–11 years. Based on participants’ input and on experts’ experience, the test versions were modified to produce pilot versions appropriate for the validation study. The pilot versions of the PKU-QOL questionnaires (Child, Adolescent, Adult and Parent) contained 43, 62, 70 and 55 items, respectively. Items were divided into sections assessing patients’ health, PKU diet and Phe-free protein supplements, patient’s/parent’s daily life with PKU, and patient’s/parent’s general feeling about PKU. The recall period focused on the past seven days for all sections except for ‘patient’s/parent’s general feeling ’ where the recall period was ‘in general’. Items had a 5-point Likert-type intensity or frequency response scale with an additional “Does not apply” or “I don’t…/My child doesn’t” response to some questions.

The final step of questionnaire development involved linguistic validation of the pilot versions of the PKU-QOL questionnaires into Turkish following a standardised and rigorous process that provides a language version that is consistent, comparable and conceptually equivalent to the original instruments [32,33]. As part of this process, interviews were conducted to test the wording of the questionnaires with Turkish patients or parents of children with PKU (Table 1).

A PKU-QOL Steering Committee (composed of ABu, AC, ABo and Pr. Peter Burgard) was convened at each key milestone of the development of the questionnaires to provide expert insight regarding: development of conceptual models, item generation and comprehension testing. The PKU-QOL Steering Committee was also convened for the design and interpretation of the validation study of the PKU-QOL questionnaire.

### Validation study design

From December 2011 to November 2012, a multicentre, prospective, non-interventional, observational study was conducted in 34 sites in France, Germany, Italy, The Netherlands, Spain, Turkey and the UK to finalise and validate the PKU-QOL questionnaires. More specifically, the primary objectives of the observational study were: (1) to define the scoring rules of the four PKU-QOL questionnaires (how items are grouped into domains); and (2) to assess the psychometric properties of these four questionnaires, namely validity (clinical validity and concurrent validity) and reliability (internal consistency reliability and test-retest reliability). Characterisation of the HRQoL of patients with PKU was a secondary objective of the study, with results presented elsewhere [34].

To be included, patients needed to be aged ≥9 years old, with confirmed diagnosis of PKU and treated for PKU with a Phe-restricted diet and/or Phe-free protein supplement and/or pharmacological therapy. To be included, parents needed to be parents of at least one patient aged <18 years old treated for PKU with a Phe-restricted diet and/or Phe-free protein supplement and/or pharmacological therapy. Patients and parents were excluded if they had a significant psychiatric disorder or conditions preventing their participation as per physician’s judgment, a history of alcohol or drug abuse in the 12 previous months or current abuse, or if they were already included in an interventional clinical trial. In addition, parents were excluded if they were diagnosed with PKU.

Three groups of patients with PKU were recruited based on age: children (9–11 years), adolescents (12–17 years) and adults (≥18 years). In addition, parents of included children and adolescents were asked to participate in the study. To supplement this study group and validate the parent questionnaire for parents of children of all ages, parents of children <9 years of age were also included, although children < 9 years were not considered able to complete the child questionnaire.

Patients/parents were asked to complete the PKU-QOL questionnaire twice (at baseline and after 2 weeks), and a generic questionnaire at baseline (Children and adolescents completed the Pediatric Quality-of-Life Inventory (PedsQL) [35], adult patients completed the Medical Outcome Survey 36 item Short Form (SF-36) [36] and Parents of patients with PKU completed the Child Health Questionnaire 28 item Parent Form (CHQ-PF28) [37]). They completed the questionnaires at home and mailed them. For each recruited patient, the physician was asked to complete a short case report form with clinical and demographic information on the patient and parent.

The study was performed in accordance with good clinical practices and in compliance with local regulatory requirements. The appropriate national authorities and institutional review boards approved the protocol before study commencement. All patients or their legally authorised representatives provided written informed consent before participation in the study.

The validation study protocol was submitted to the Conseil National de l’ordre de Médecins (CNOM) in France, to the Ethik-Kommission an der Medizinischen Fakultät der Universität Leipzig, Ethikkommission der Medizinischen Fakultät Heidelberg, Ethik-Kommission der MHH, Ethik-Kommission des Fachbereichs Medizin der Johann Wolfgang Goethe- Universität, Ethik-Kommission der Ärztekammer Westfalen-Lippe und der medizinischen Fakultät der Westfälischen Wilhelms Universität Münster and Ethik-Kommission bei der Landesärztekammer Baden-Württemberg in Germany, to the Comitato Etico della Azienda Ospedaliero-Universitaria di Bologna, Comitato Etico per la Sperimentazione Clincal della Provincia di Vicenza, Comitato Etico per la Sperimentazione della Azienda Ospedaliera di Padova, Comitato Etico della ASL NA/1 di Napoli, Comitato di Etica dell 'IRCCS Istituto Giannina Gaslini de Genova' and Comitato Etico Dell 'Azienda Policlinico Umberto I Di Roma' in Italy, to the Academisch Medisch Centrum Amsterdam, Universitair Medisch Centrum Groningen and Academisch Ziekenhuis Maastricht (AZM) in the Netherlands, to CCAA Andalucía, CCAA Galicia, CCAA Aragón, CCAA Baleares, CCAA Pais Vasco and H. Univ Virgen del Rocio in Spain, to Istanbul University Cerrahpasa Medical Faculty Ethic Committee in Turkey, to R&D of Glasgow Royal Infirmary, R&D of Central Manchester University Hospitals NHS Foundation Trust, R&D of University Hospitals Bristol and Guy's & St Thomas' Hospital NHS Foundation Trust in the UK.

### Statistical analyses

#### Scaling structure and the scoring rules of the four PKU-QOL questionnaires

Item selection and creation of the scoring method were based on the quality of completion of the items, the distribution of the responses, and the initial hypothesised structure of the questionnaires.

Multi-trait analysis [38] was used to test the association between single items and the grouping of items into domains. This method is based on the analysis of the correlations between each item and each subscale according to the following principles: each item should be strongly correlated with its own scale –correlation coefficient greater than 0.4 are expected [39] – (*item convergent criterion*) and should be more correlated with its own scale than with others (*item discriminant criterion*). Iterative applications of the multi-trait analysis were performed with the following constraints: the structure should accurately reflect conceptual preconceptions and be kept as similar as possible across the four questionnaires, and the different areas explored by the questionnaire (i.e. PKU symptoms, impact of PKU and its management, dietary protein restrictions, administration of Phe-free protein supplements) should be kept separate.

#### Assessment of psychometric properties

The psychometric validation included assessment of reliability (internal consistency reliability and test-retest reliability) and validity (concurrent validity and clinical validity). Internal consistency reliability was estimated using Cronbach’s alpha [40]. Test-retest reliability is defined as the extent to which the questionnaire leads to the same results on repeated assessments over short periods of time. The PKU-QOL questionnaires were administered twice (i.e. baseline and Week 2) to allow the assessment of test-retest reliability which was measured by calculating the Intraclass Correlation Coefficient (ICC) [41]. A recommended threshold for reliability coefficients (i.e. Cronbach’s alpha or ICC) is 0.7 [42].

Validity is defined as the accuracy with which a measurement tool measures the concept it is intended to measure. Concurrent validity was evaluated by investigating the association between PKU-QOL scores and scores of generic HRQoL questionnaires corresponding to each age group: the Pediatric Quality-of-Life Inventory (PedsQL) for children and adolescents [35], the 36-item Short Form (SF-36) Health Survey [36] for adults, and the Child Health Questionnaire–Parent Form 28 (CHQ-PF28) [37] for parents, using Spearman correlation coefficients. The hypothesis was that domains measuring related concepts have high correlation levels while domains measuring different concepts have low correlations. For clinical validity, the associations between PKU-QOL scores with severity of PKU (Phe levels at diagnosis), using a t-test, and with overall assessment of patient’s health status as rated by the investigator who recruited the patient (i.e., “In general, how would you rate the overall health status of your patient?” Poor/Fair/Good/Very good/Excellent), using an analysis of variance (ANOVA), were observed. The patients with worse health status or more severe PKU were expected to have worse HRQoL.

## Results

### Population characteristics in the validation study

#### Evaluable population

Of the 617 subjects recruited in the study, 559 returned a PKU-QOL questionnaire and were used in the analyses: 306 patients with PKU and 253 parents of patients with PKU. The patient population included 92 children aged 9 to 11 years, 110 adolescents aged 12 to 17 years and 104 adults older than 18 years of age. The Parent population included the parents of 201 subjects from the child or adolescent population, and 52 additional parents of children younger than 8 years old. See Table 1 for a repartition by age group and country.

#### Demographics

Patients who completed the PKU-QOL were aged between 9 and 45 years (Table 2). The age distribution in the child and adolescent populations covered the full age range (9–11 and 12–17 years), with a mean age in the middle of this range (9.8 years for children and 14.5 years for adolescents). The mean age of adult PKU patients was 25.8 years. Parents were between 24 and 66 years old, with a mean age of 41.6 years, and their children had a mean age of 10.7 years. There were about as many male as female patients in both the child and adolescent groups (46.7% and 50.9% males, respectively), whereas there were more females than males in the adult and parent groups (63.5% and 72.7% females, respectively).

**Table 2:** **Demographic characteristics in the patient and parent populations of the validation study**

		**Child (9–11 yo) evaluable population**	**Adolescent (12–17 yo) evaluable population**	**Adult (>18 yo) evaluable population**	**Parent evaluable population**	**Children (0–18 yo) of the parent population**
		**(n = 92)**	**(n = 110)**	**(n = 104)**	**(n = 253)**	**(n = 253)**
**Age (years)**	**n (missing)**	90 (2)	110 (0)	101 (3)	244 (9)	251 (2)
	**Mean (SD)**	9.8 (0.8)	14.5 (1.6)	25.8 (6.6)	41.6 (6.5)	10.7 (4.2)
	**Min – Max**	9.0 **–** 11.0	12.0 **–** 17.0	18.0 **–** 45.0	24.0 **–** 66.0	0.0 **–** 17.0
**Sex**	**Male, n (%)**	43 (46.7)	56 (50.9)	38 (36.5)	69 (27.3)	126 (49.8)
**PKU severity**	Classical PKU*, n (%)	66 (71.7)	75 (68.2)	67 (64.4)	-	172 (68.0)

SD: standard deviation.

*Classical PKU defined as Phe level at diagnosis >1200 μmol/L.

More than two-thirds of the patients (68%) had classic PKU (characterized by blood Phe level at diagnosis >1200 μmol/L). This proportion of patients with classic PKU was fairly stable across all age groups.

Almost all patients were following dietary restriction (94.1%) and were taking Phe-free protein supplements (89.2%), and 22.5% were receiving pharmacological therapy, tetrahydrobiopterin (BH_4_).

### Scaling structure and scoring rules

#### Return rate

The return rate of the PKU-QOL questionnaires was 90% at baseline and 77% at Week 2, indicating a good acceptability of the questionnaire by patients and their parents. The return rate was notably lower at both time points (81% [baseline] and 63% [Week 2]) for parents of young children (8 years old or younger).

#### Quality of completion

On average, there were 1.6, 3.0, 1.4 and 2.4 missing items in the Child PKU-QOL, Adolescent PKU-QOL, Adult PKU-QOL and Parent PKU-QOL, respectively. Given the relatively high number of items in the questionnaires, this represented a small number of missing data (<5% of the items for all versions). The items that had a higher level of missing data were those asking about the ‘attribution’ of a symptom to PKU (e.g. *‘If you had headaches, do you think it was related to PKU?’*). These were systematically missing for more than 10% of the patients. This could be due to a difficulty in understanding those questions, or in attributing a symptom to the disease.

### Distribution of responses

The distribution of the responses was skewed towards the most positive response options for a large majority of items. The items with the most severely skewed response distribution were listed and reviewed by the PKU-QOL Steering Committee to determine whether they were non-informative and could be deleted from the questionnaire or were considered a key concept that should be retained in the final questionnaire. Eight items with severely skewed distribution were identified in the Child PKU-QOL questionnaire, seven in the Adolescent PKU-QOL, 14 in the Adult PKU-QOL and two in the parent versions. Among these, three were deleted after PKU-QOL Steering Committee appraisal in the Child PKU-QOL questionnaire (‘*Nausea’; ‘Participating in activities at school’; ‘Difficulty to do other things due to burden of care for PKU’*), four in the Adolescent PKU-QOL questionnaire (‘*Nausea’; ‘Participating in activities at school’; ‘Difficulty to do other things due to burden of care for PKU’, ‘Difficulty starting romantic relationships’*), five in the Adult PKU-QOL questionnaire (‘*Nausea’, ‘Participating in activities at school’, ‘Difficulty to do other things due to burden of care for PKU’, ‘Difficulty starting romantic relationships, ‘missing supplements because of college/university constraints’*) and one in the Parent PKU-QOL questionnaire (‘*Nausea*’).

### Scaling structure

The four questionnaires were structured in four modules: PKU symptoms, PKU in general, administration of Phe-free protein supplements and dietary protein restriction (see Table 3 for the final scaling structure of the four questionnaires). Iterative multitrait analyses led to the grouping of items in domains within each module. Results of the final multitrait analyses for each module of each version of the PKU-QOL questionnaire are provided as Additional file 1.

**Table 3:** **Final scaling/scoring structure of the four PKU-QOL questionnaires**

**Modules**	**Domain scores**	**PKU-QOL items (for multi-item scores)**	**Child PKU-QOL (40 items)**	**Adolescent PKU-QOL (58 items)**	**Adult PKU-QOL (65 items)**	**Parent PKU-QOL (54 items)**
**PKU symptoms**	Self-health rated status			*√*	*√*	*√*
	Headaches		*√*	*√*	*√*	*√*
	Stomach aches		*√*	*√*	*√*	*√*
	Tiredness		*√*	*√*	*√*	*√*
	Lack of concentration		*√*	*√*	*√*	*√*
	Slow thinking		*√*	*√*	*√*	*√*
	Trembling hands				*√*	
	Irritability		*√*	*√*	*√*	*√*
	Aggressiveness		*√*	*√*	*√*	*√*
	Moodiness		*√*	*√*	*√*	*√*
	Sadness		*√*	*√*	*√*	*√*
	Anxiety		*√*	*√*	*√*	*√*
**PKU in general**	Emotional impact of PKU	Unfairness having PKU	*√*	*√*	*√*	*√*
		Worries about the future		*√*	*√*	*√*
		Worries about future children		*√*	*√*	*√*
		Disease acceptance	*√*	*√*	*√*	*√*
		Self-esteem	*√*	*√*	*√*	*√*
	Practical impact of PKU	Burden of care for PKU	*√*	*√*	*√*	*√*
		Burden of physician visits	*√*	*√*	*√*	*√*
		Maintaining activity at work		*√*	*√*	*√*
		Time in administrative tasks			*√*	*√*
		Missing work				*√*
		Difficulty to do other things due to the burden of care for PKU				*√*
	Social impact of PKU	Explain situation to others	√	√	√	√
		Discussing PKU within family	√	√	√	√
		Difficulty making friends	√	√	√	√
		Impact on relationship with partner			*√*	*√*
		Maintaining friendship				*√*
		Impact of PKU on child’s siblings				*√*
	Overall impact of PKU*	*Items from Emotional, practical and social impact of PKU*	*√*	*√*	*√*	*√*
	Anxiety – blood tests	Anxiety having blood test in the arm	*√*	*√*	*√*	*√*
		Anxiety having blood test in the finger	*√*	*√*	*√*	*√*
	Impact of child anxiety – blood tests	Impact of child anxiety of blood test in the arm on parent				*√*
		Impact of child anxiety of blood test in the finger on parent				*√*
	Anxiety – Phe levels		*√*	*√*	*√*	*√*
	Anxiety – Phe levels during pregnancy				*√*	
	Financial impact of PKU				*√*	*√*
	Information on PKU				*√*	*√*
**Administration of Phe-free protein supplements**	Adherence to Phe-free protein supplements	Frequency of supplement intake	*√*	*√*	*√*	
		Adherence/compliance to supplement	*√*	*√*	*√*	*√*
		Missing supplements because of school		*√*		
		Missing supplements because of work constraints		*√*	*√*	
	Guilt if poor adherence to Phe-free protein supplements		*√*	*√*	*√*	*√*
	Impact of Phe-free protein supplements on family		*√*	*√*	*√*	*√*
	Practical impact of Phe-free protein supplements	Embarrassment/shame taking supplements	*√*	*√*	*√*	
		Lack of spontaneity/freedom due to supplements		*√*	*√*	*√*
		Difficulty eating out due to supplements		*√*	*√*	*√*
		Difficulty travelling, transporting supplements for special event situations		*√*	*√*	*√*
	Taste - Phe-free protein supplements		*√*	*√*	*√*	
	Management of Phe-free protein supplements					*√*
**Dietary protein restriction**	Food temptations	Temptation/adherence	*√*	*√*	*√*	
		Temptation not emotionally	*√*	*√*	*√*	
	Adherence to diet	Adherence/compliance to the diet	*√*	*√*	*√*	*√*
		Eat forbidden things secretly	*√*	*√*	*√*	
		Change in diet because of school/college/university constraints		*√*	*√*	
		Change in diet because of work constraints		*√*	*√*	
		Eat forbidden food intentionally				
	Management of diet	Difficulty monitoring dietary protein restriction				*√*
		Difficulty monitoring the amount of calories				*√*
		Sadness to forbid food to child				*√*
		Worry that child eats things secretly				*√*
		Hard when child has to follow diet in front of others				*√*
		Impact of diet on relationship with child				*√*
	Practical impact of diet	Burden of weighing/estimating quantity of protein in food		*√*	*√*	*√*
		Lack of spontaneity/freedom due to PKU diet		*√*	*√*	*√*
		Difficulty eating out due to PKU diet		*√*	*√*	*√*
		Need to plan meals in advance		*√*	*√*	*√*
		Time-consuming aspects of the diet		*√*	*√*	*√*
		Complexity cooking		*√*	*√*	*√*
		Difficulty travelling, transporting PKU food for special event situations		*√*	*√*	*√*
	Social impact of diet	Feeling different because of diet	*√*	*√*	*√*	
		Relationship within family	*√*	*√*	*√*	
		Temptation emotionally	*√*	*√*	*√*	
		Embarrassment/shame following diet	*√*	*√*	*√*	
		Isolation because of diet	*√*	*√*	*√*	
		Cooking for others			*√*	
	Overall impact of diet*	*Items from practical and social impact of dietary protein restriction*		*√*	*√*	
	Overall difficulty following diet		*√*	*√*	*√*	
	Guilt if diet not followed		*√*	*√*	*√*	*√*
	Taste of low-protein food		*√*	*√*	*√*	
	Food enjoyment		*√*	*√*	*√*	*√*

In bold*: Overall multi-item scores including items from different domains.

Phe: phenylalanine; PKU: phenylketonuria.

The ‘PKU symptoms’ module consists of single-item symptom scores.

The domains related to the ‘PKU in general’ module include the following PKU impact scores: practical, social, emotional and overall impact of PKU. In addition, additional specific scores were created: anxiety due to blood tests, and anxiety due to high blood Phe levels. The adult and parent versions included single-item scores assessing the level of information on PKU and the financial impact of PKU, and the parent version included an extra score for the impact on the parent owing to the child’s anxiety towards blood tests.

The domains related to the module ’administration of Phe-free protein supplements’ include scores of adherence to Phe-free protein supplements, score on guilt due to poor adherence to Phe-free protein supplements, and scores of impact of Phe-free protein supplements on daily life and family.

Finally, the domains related to the ‘dietary protein-restrictions’ module included scores on food temptations (except for parents), adherence to dietary protein restrictions, overall difficulty following dietary restrictions, guilt if the diet is not followed, social impact of the diet, practical impact of the diet (except for children), food enjoyment and taste of specialty low-protein food products. An overall score gathering the two impact scores was also created.

### Scoring rules

For each item of the questionnaire, an item score ranging from 0 to 4 was obtained based on the response of the patient. Then, domain scores were calculated by summing the item scores and applying linear transformation to the sum to have all domain scores ranging from 0 to 100:

$$ \mathrm{domain}\ \mathrm{s}\mathrm{core}=\frac{\mathrm{Sum}\ \mathrm{o}\mathrm{f}\ \mathrm{item}\ \mathrm{s}\mathrm{core}\mathrm{s}\ \mathrm{within}\ \mathrm{the}\ \mathrm{domain}}{\mathrm{Number}\ \mathrm{o}\mathrm{f}\ \mathrm{n}\mathrm{o}\mathrm{n}\ \mathrm{missing}\ \mathrm{item}\ \mathrm{s}\mathrm{core}\mathrm{s}\ \mathrm{within}\ \mathrm{the}\ \mathrm{domain}}*25 $$

Each domain score is calculated only if at least 70% of the items of the domain have been completed; otherwise the domain score was set as missing.

The following interpretation rules were applied for all domain scores in a range from 0 to 100:

- for symptom scores, a higher score is associated with more frequent symptoms,
- for adherence scores, a higher score is associated with a poorer adherence,
- for other scores, a higher score is associated with a greater impact.

### Psychometric properties

#### Validity

##### Concurrent validity

Overall, the pattern of correlation between the scores of the PKU-QOL questionnaires and scores of the generic instruments was meaningful: the highest correlation coefficients were observed between scores supposed to assess close concepts. In particular, the ‘Emotional impact of PKU’ scores were well correlated with generic scores of ‘Emotional functioning’ (as measured by the PedsQL) in children and adolescents (see Table 4) and with the generic score of ‘Parental emotional impact’ in parents (as measured by the CHQ-PF28) (see Table 5). In adults, the PKU-QOL score ‘Overall impact of PKU’ was correlated with the ‘General health’ score of the SF-36 (see Table 6).

**Table 4:** **Spearman correlation coefficients between child PKU-QOL and adolescent PKU-QOL and PedsQL scores**

**PKU-QOL scores**	**Child evaluable population (n = 92)**				**Adolescent evaluable population (n = 110)**			
	**EF**	**PF**	**SoF**	**ScF**	**EF**	**PF**	**SoF**	**ScF**
**Self-rated health status**	-	-	-	-	−0.26	−0.23	−0.22	−0.16
**Headaches**	−0.07	−0.25	−0.05	−0.22	−0.23	−0.28	−0.22	−0.26
**Stomach aches**	−0.33	−0.06	−0.06	−0.17	−0.16	−0.23	−0.25	−0.11
**Tiredness**	−0.13	−0.34	−0.21	−0.30	−0.23	−0.22	−0.15	−0.18
**Angry**	−0.38	−0.36	−0.24	−0.30	−0.30	−0.08	−0.27	−0.08
**Aggressiveness**	−0.08	−0.13	−0.00	−0.10	−0.31	−0.20	−0.21	−0.31
**Moodiness**	−0.13	−0.26	−0.11	−0.25	−0.28	−0.10	−0.10	−0.27
**Sadness**	**−0.41**	−0.33	−0.33	−0.23	−0.31	−0.17	−0.23	−0.16
**Anxiety**	−0.26	−0.05	−0.31	−0.12	−0.27	−0.18	−0.27	−0.10
**Lack of concentration**	−0.15	−0.32	−0.33	−0.36	−0.21	−0.19	−0.27	**−0.48**
**Slow thinking**	−0.24	**−0.45**	−0.37	**−0.43**	−0.32	**−0.42**	**−0.42**	−0.36
**Emotional impact of PKU**	**−0.45**	−0.22	−0.28	−0.33	**−0.47**	−0.19	−0.31	−0.28
**Practical impact of PKU**	−0.40	−0.24	−0.25	−0.09	−0.29	−0.19	−0.30	−0.28
**Social impact of PKU**	−0.39	−0.38	**−0.41**	−0.20	−0.35	−0.10	−0.20	−0.15
**Overall impact of PKU**	**−0.54**	−0.35	**−0.41**	−0.30	**−0.49**	−0.21	−0.31	−0.31
**Anxiety – blood test**	−0.13	−0.05	−0.04	0.05	−0.11	−0.16	−0.05	−0.03
**Anxiety – blood Phe levels**	**−0.42**	−0.25	−0.13	−0.37	−0.37	−0.14	−0.18	−0.25
**Adherence to Phe-free protein supplements**	−0.12	−0.11	0.00	−0.11	−0.13	−0.23	−0.24	−0.26
**Practical impact of Phe-free protein supplements**	−0.23	−0.18	−0.21	−0.16	**−0.43**	−0.31	−0.36	−0.25
**Guilt if poor adherence to Phe-free protein supplements**	0.14	0.09	0.01	0.18	−0.11	0.11	0.04	0.08
**Relationships within family because of Phe-free protein supplements**	−0.30	−0.18	−0.18	−0.11	−0.16	−0.11	−0.14	−0.37
**Taste – Phe-free protein supplements**	−0.28	−0.06	−0.10	−0.23	−0.06	0.00	0.08	−0.17
**Food temptations**	−0.32	−0.29	−0.35	−0.14	−0.36	−0.10	−0.24	−0.18
**Adherence to dietary protein – restriction**	−0.03	−0.14	−0.01	−0.11	−0.39	−0.09	−0.29	−0.18
**Social impact of dietary protein restriction**	−0.40	**−0.45**	−0.40	**−0.47**	−0.37	−0.19	−0.34	−0.34
**Practical impact of dietary protein restriction**	-	**-**	-	**-**	**−0.44**	−0.24	−0.30	−0.40
**Overall impact of dietary protein restriction**	-	**-**	-	**-**	**−0.45**	−0.27	−0.33	−0.35
**Taste – specialty low-protein food**	−0.12	0.08	0.13	−0.01	−0.19	−0.03	−0.09	−0.15
**Food enjoyment**	−0.19	−0.23	−0.26	−0.11	0.04	0.03	−0.08	−0.08
**Guilt if dietary protein restriction not followed**	0.01	−0.08	−0.10	−0.03	−0.24	−0.03	−0.16	−0.03
**Overall difficulty following dietary protein restriction**	−0.32	−0.23	−0.24	−0.21	−0.37	−0.16	−0.19	−0.32

PedsQL: Pediatric Quality-of-Life Inventory; Phe: phenylalanine; PKU: phenylketonuria; QOL: quality of life; EF: Emotional functioning; PF: Physical functioning; SoF: Social functioning; ScF: School functioning.

Moderate correlations (>0.4) are shown in bold.

**Table 5:** **Spearman correlation coefficients between Parent PKU-QOL and CHQ-PF28 scores in the parent evaluable population (n = 253)**

	**CHQ-PF28 scores**												
**PKU-QOL scores**	**PF**	**RP**	**GH**	**BP**	**PiT**	**PiE**	**RE**	**SE**	**MH**	**Be**	**FA**	**FC**	**CH**
**Child health status**	−0.24	−0.24	**−0.43**	−0.26	−0.34	−0.39	−0.09	−0.37	−0.23	−0.37	−0.37	−0.26	0.15
**Headaches**	−0.06	−0.10	−0.29	−0.32	−0.18	−0.21	−0.12	−0.25	−0.29	−0.25	−0.22	−0.16	0.16
**Stomach aches**	−0.11	−0.06	−0.18	−0.40	−0.11	−0.23	−0.02	−0.08	−0.27	−0.15	−0.17	−0.03	0.17
**Tiredness**	−0.24	−0.21	−0.28	−0.28	−0.34	−0.36	−0.19	−0.26	−0.35	−0.28	−0.23	−0.11	−0.00
**Lack of concentration**	−0.10	−0.07	−0.23	−0.23	−0.20	−0.22	−0.28	−0.36	−0.33	**−0.57**	−0.22	−0.17	−0.06
**Slow thinking**	−0.16	−0.10	−0.29	−0.20	−0.20	−0.23	−0.40	−0.37	−0.36	**−0.47**	−0.26	−0.17	0.03
**Irritability**	−0.12	−0.09	−0.28	−0.16	−0.28	−0.36	−0.23	**−0.42**	**−0.45**	**−0.54**	−0.27	−0.29	0.03
**Aggressiveness**	−0.16	−0.22	−0.19	−0.12	−0.26	−0.28	−0.31	−0.31	−0.37	**−0.49**	−0.28	−0.24	−0.09
**Moodiness**	−0.14	−0.18	−0.21	−0.26	−0.32	**−0.43**	−0.31	−0.40	**−0.43**	**−0.53**	−0.37	−0.24	−0.07
**Sadness**	−0.16	−0.10	−0.25	−0.32	−0.36	−0.30	−0.36	−0.38	**−0.43**	−0.35	−0.28	−0.16	0.03
**Anxiety**	−0.18	−0.15	−0.24	−0.18	−0.24	−0.16	−0.34	−0.39	**−0.41**	−0.20	−0.21	−0.21	0.01
**Emotional impact of PKU**	−0.14	−0.15	−0.33	−0.21	−0.23	−0.40	−0.04	−0.22	−0.29	−0.26	−0.29	−0.27	−0.03
**Practical impact of PKU**	−0.20	−0.18	−0.35	−0.21	−0.33	−0.39	−0.15	−0.24	−0.37	−0.34	**−0.42**	−0.25	0.07
**Social impact of PKU**	−0.20	−0.18	−0.29	−0.24	−0.26	−0.35	−0.20	−0.25	−0.33	−0.31	−0.37	−0.36	−0.03
**Overall impact of PKU**	−0.21	−0.20	−0.39	−0.26	−0.31	**−0.46**	−0.12	−0.29	−0.37	−0.35	**−0.44**	−0.34	−0.02
**Anxiety – blood test**	−0.15	−0.13	−0.14	−0.05	−0.21	−0.22	−0.03	−0.01	−0.15	−0.08	−0.27	−0.06	0.08
**Impact of anxiety – blood test**	−0.19	−0.20	−0.24	−0.10	−0.26	−0.26	−0.09	0.02	−0.14	−0.05	−0.29	−0.03	0.26
**Anxiety – blood Phe levels**	−0.06	−0.06	−0.25	−0.13	−0.13	**−0.42**	−0.09	−0.06	−0.28	−0.24	−0.19	−0.09	0.05
**Financial impact of PKU**	−0.12	−0.05	−0.20	−0.19	−0.19	−0.29	−0.06	−0.05	−0.15	−0.26	−0.24	−0.20	0.03
**Information on PKU**	−0.14	−0.19	−0.25	−0.26	−0.14	−0.24	−0.14	−0.30	−0.16	−0.24	−0.19	−0.27	0.00
**Adherence to Phe-free protein supplements**	−0.09	−0.16	−0.10	−0.13	−0.11	−0.08	−0.22	−0.24	−0.21	−0.28	−0.12	−0.07	0.03
**Management of Phe-free protein supplements**	−0.03	−0.12	−0.09	−0.10	−0.28	−0.21	−0.05	−0.07	−0.18	−0.28	−0.22	−0.14	−0.06
**Practical impact of Phe-free protein supplements**	−0.15	−0.23	−0.19	−0.12	−0.17	−0.18	−0.04	−0.06	−0.25	−0.10	−0.38	−0.26	−0.03
**Guilt if poor adherence to Phe-free protein supplements**	−0.14	−0.07	−0.27	−0.09	−0.10	−0.27	−0.11	0.08	−0.20	−0.10	−0.14	−0.05	0.15
**Relationships within family because of Phe-free protein supplements**	−0.17	−0.21	−0.15	−0.26	−0.33	−0.22	−0.15	−0.16	−0.24	−0.34	−0.32	−0.23	−0.02
**Adherence to dietary protein restriction**	−0.04	−0.09	−0.17	−0.08	−0.19	−0.11	−0.14	−0.15	−0.09	−0.24	−0.16	−0.10	0.07
**Management of dietary protein restriction**	−0.12	−0.16	−0.26	−0.25	−0.30	−0.40	−0.24	−0.34	−0.35	**−0.49**	**−0.41**	−0.29	−0.04
**Practical impact of dietary protein restriction**	−0.15	−0.24	−0.24	−0.28	−0.30	−0.33	−0.10	−0.30	−0.32	−0.33	**−0.46**	−0.39	−0.14
**Food enjoyment**	−0.11	−0.06	−0.19	−0.28	−0.21	−0.14	−0.07	−0.26	−0.27	−0.24	−0.28	−0.28	0.07
**Guilt if dietary protein restriction not followed**	−0.15	−0.04	−0.15	−0.07	−0.05	−0.31	−0.12	−0.00	−0.15	−0.07	−0.12	−0.04	0.04

CHQ-PF: Child Health Questionnaire–Parent Form; Phe: phenylalanine; PKU: phenylketonuria; QOL: quality of life; PF: Physical functioning; RP: Role/Social Physical; GH: General health; BP: Bodily Pain; PiT: Parent impact Time; PiE: Parent impact Emotional; RE: Role/Social Emotional; SE: Self-Esteem; MH: Mental Health; Be: Behaviour; FA: Family Activity; FC: Family Cohesion; CH: Change in Health.

Moderate correlations (>0.4) are shown in bold.

**Table 6:** **Spearman correlation coefficients between Adult PKU-QOL and SF-36 scores in the adult evaluable population (n = 104)**

**PKU-QOL scores**	**SF-36 scores**							
	**PF**	**RP**	**BP**	**GH**	**VT**	**SF**	**RE**	**MH**
**Self-rated health status**	−0.37	−0.22	−0.30	**−0.58**	**−0.57**	−0.36	−0.29	**−0.50**
**Headaches**	−0.20	−0.26	−0.39	−0.31	−0.29	**−0.41**	−0.30	−0.35
**Stomach aches**	−0.17	−0.19	−0.40	−0.30	−0.34	−0.26	−0.33	−0.37
**Tiredness**	−0.28	−0.34	−0.30	−0.40	**−0.52**	−0.40	−0.32	−0.39
**Lack of concentration**	−0.25	**−0.59**	−0.37	−0.40	**−0.52**	**−0.51**	**−0.56**	**−0.60**
**Slow thinking**	−0.27	−0.36	−0.29	−0.36	−0.32	**−0.44**	**−0.47**	**−0.43**
**Trembling hands**	−0.16	−0.21	−0.19	−0.31	−0.31	−0.19	−0.25	−0.28
**Irritability**	−0.23	**−0.44**	−0.32	−0.29	−0.20	−0.31	**−0.40**	−0.39
**Aggressiveness**	−0.22	−0.36	−0.21	−0.24	−0.22	−0.31	−0.31	−0.24
**Moodiness**	−0.21	−0.40	**−0.45**	−0.38	**−0.41**	**−0.54**	**−0.50**	**−0.53**
**Sadness**	−0.21	**−0.44**	**−0.48**	**−0.49**	−0.39	**−0.61**	**−0.56**	**−0.66**
**Anxiety**	−0.16	−0.31	−0.26	−0.31	−0.29	−0.37	**−0.43**	**−0.47**
**Emotional impact of PKU**	**−0.41**	−0.31	−0.35	**−0.43**	−0.34	−0.35	−0.31	−0.39
**Practical impact of PKU**	−0.05	−0.38	−0.18	−0.38	−0.38	−0.38	**−0.47**	−0.38
**Social impact of PKU**	−0.34	−0.32	−0.30	−0.40	−0.30	**−0.48**	−0.35	−0.39
**Overall impact of PKU**	−0.27	**−0.42**	**−0.42**	**−0.53**	−0.32	**−0.53**	**−0.45**	**−0.48**
**Anxiety – blood test**	0.05	−0.03	−0.27	−0.16	−0.12	−0.32	−0.22	−0.25
**Anxiety – Phe levels**	−0.19	−0.11	−0.21	−0.28	−0.19	−0.25	−0.17	−0.15
**Anxiety – Phe levels during pregnancy**	−0.11	0.11	−0.03	−0.02	−0.00	−0.05	0.06	−0.13
**Financial impact of PKU**	−0.11	−0.05	−0.05	0.14	0.03	0.01	0.03	0.08
**Information on PKU**	−0.08	−0.11	−0.15	−0.12	−0.08	−0.17	−0.22	−0.19
**Adherence to Phe-free protein supplements**	−0.10	−0.28	−0.25	−0.14	−0.29	−0.35	−0.38	−0.18
**Practical impact of Phe-free protein supplements**	−0.13	−0.13	−0.11	**−0.41**	**−0.43**	−0.33	−0.25	−0.37
**Guilt if poor adherence to Phe-free protein supplements**	−0.12	−0.16	−0.23	−0.22	−0.13	−0.30	−0.13	−0.23
**Relationships within family because of Phe-free protein supplements**	−0.02	−0.01	−0.20	−0.15	−0.13	−0.13	0.01	−0.09
**Taste – Phe-free protein supplements**	0.07	0.12	0.04	−0.24	−0.25	−0.18	−0.00	0.00
**Food temptations**	−0.14	−0.40	−0.19	−0.21	−0.25	−0.37	−0.31	−0.33
**Adherence to dietary protein restriction**	−0.03	−0.29	−0.13	−0.06	−0.17	−0.24	−0.29	−0.29
**Social impact of dietary protein restriction**	−0.12	−0.40	−0.20	**−0.43**	**−0.44**	**−0.50**	**−0.50**	**−0.49**
**Practical impact of dietary protein restriction**	−0.21	−0.25	−0.11	−0.38	**−0.59**	**−0.44**	−0.39	**−0.46**
**Overall impact of dietary protein restriction**	−0.23	−0.33	−0.17	**−0.45**	**−0.57**	**−0.54**	**−0.44**	**−0.49**
**Taste – specialty low-protein food**	−0.09	−0.31	−0.27	−0.33	−0.25	−0.34	**−0.41**	−0.33
**Food enjoyment**	−0.19	−0.28	−0.16	−0.36	−0.34	**−0.41**	−0.38	−0.29
**Guilt if dietary protein restriction not followed**	−0.17	−0.15	−0.21	−0.30	−0.10	−0.33	−0.12	−0.16
**Overall difficulty following dietary protein restriction**	−0.33	**−0.47**	−0.21	−0.38	**−0.46**	**−0.46**	−0.34	−0.33

Phe: phenylalanine; PKU: phenylketonuria; QOL: quality of life; SF-36: 36-item Short Form; PF: Physical functioning; RP: Role Physical; BP: Bodily Pain; GH: General health; VT: Vitality; SF: Social Functioning; RE: Role Emotional; MH: Mental Health.

Correlations between Adult PKU-QOL and SF-36 scores were low to moderate. Moderate correlations (>0.4) are shown in bold.

For all PKU-QOL questionnaires, the disease symptom scores were among the scores with the highest correlations with generic HRQoL measures. This could be explained by the natural fairly direct relationship between the symptoms of a chronic disease like PKU and the HRQoL of patients coping with this disease. Conversely, the scores related to Phe-free protein supplements or dietary restrictions tap into domains of patients’ life that cannot be captured by the generic measures. This also justifies the need for specific measures of PKU to accurately reflect the impact of the disease.

##### Clinical validity

Comparison of the PKU-QOL scores according to overall health status as rated by the investigator

PKU-QOL symptom scores and scores assessing the impact of PKU, which could be assumed to be related to a patient’s overall health status, tended to have lower medians in patients with better rating of health status. For example, the median ‘Overall impact of PKU’ score for adults with an ‘excellent’ health status was 18 while it was 27 for adults with ‘very good’ health status and 40 for those with a ‘good’ health status. In adolescents, the median ‘Overall impact of PKU’ score was 18 for patients with an ‘excellent’ health, 20 for those with a ‘very good’ health and 25 for those with a ‘good’ health. In children, the median ‘Overall impact of PKU’ score was 17 for patients with an ‘excellent’ health, 19 for those with a ‘very good’ health and 28 for those with a ‘good’ health. On the other hand, domain scores which were not expected to be directly related with global health status (scores related to the impact of Phe-free protein supplement intake or dietary protein restriction) showed little association with patient’s health status as assessed by the clinician.

Comparison of the PKU-QOL scores according to severity of PKU

No clear association between the PKU-QOL domain scores assessing the symptoms of PKU or the impact of PKU and the severity of PKU (as defined by blood Phe level at diagnosis) were observed. A consistent pattern emerged in the association between scores assessing the impact of Phe-free protein supplement and the severity of PKU: patients with classical PKU reporting a higher impact of Phe-free protein supplements. In particular, adolescent and adult patients with mild/moderate PKU had a median ‘Practical impact of supplements’ of respectively 6 and 13 while for those with classical PKU median was 19 in both groups. PKU-QOL scores assessing the aspects related to the diet were associated to the severity of PKU in adolescents and children: adolescents and children with classical PKU had a slightly poorer adherence to their diet and higher impact (social and emotional). However, this association pattern was not found in adults.

Detailed results of the clinical validity for the Child, Adolescent, Adult, and Parent PKU-QOL questionnaires are provided as Additional files 2, 3, 4, and 5, respectively.

### Reliability

#### Internal consistency reliability

Internal consistency reliability of the majority of multi-item scores in adults, adolescents and parents was acceptable (above the threshold value of 0.70), even if some scores were not fully satisfactory (practical impact of PKU: Adolescent, α = 0.47; Adult, α = 0.50; and social impact of PKU: Adolescent, α = 0.45; Adult, α = 0.63). Reliability coefficients of the Child PKU-QOL scores were below the standards generally used. See Tables 7 and 8.

**Table 7:** **Reliability coefficients of the Child, Adolescent and Adult PKU-QOL questionnaires at baseline**

	**Child PKU-QOL (n = 92)**		**Adolescent PKU-QOL (n = 110)**		**Adult PKU-QOL (n = 104)**	
	**Cronbach** α	**ICC**	**Cronbach** α	**ICC**	**Cronbach** α	**ICC**
**Self-rated health status**	-	-	-	0.49	-	0.67
**Headaches**	-	0.37	-	0.52	-	0.59
**Stomach aches**	-	0.33	-	0.34	-	0.61
**Tiredness**	-	0.27	-	0.51	-	0.62
**Irritability/Anger**	-	0.45	-	0.53	-	0.42
**Aggressiveness**	-	0.44	-	0.64	-	0.53
**Moodiness**	-	0.47	-	0.59	-	0.66
**Sadness**	-	0.35	-	0.52	-	0.57
**Anxiety**	-	0.44	-	**0.70**	-	0.60
**Lack of concentration**	-	0.47	-	0.60	-	0.69
**Slow thinking**	-	0.55	-	0.61	-	0.63
**Trembling hands**	-	-	-	-	-	**0.75**
**Emotional impact of PKU**	0.37	0.60	**0.70**	**0.83**	**0.71**	**0.84**
**Practical impact of PKU**	0.43	0.45	0.47	**0.75**	0.50	**0.70**
**Social impact of PKU**	0.59	**0.70**	0.45	**0.83**	0.63	**0.77**
**Overall impact of PKU**	0.69	0.67	**0.80**	**0.88**	**0.79**	**0.84**
**Anxiety – blood test**	0.26	0.64	0.61	**0.95**	**0.77**	**0.87**
**Anxiety – blood Phe levels**	-	0.64	-	**0.76**	-	0.63
**Anxiety – blood Phe levels during pregnancy**	-	-	-	-	-	**0.71**
**Financial impact of PKU**	-	-	-	-	-	0.65
**Information on PKU**	-	-	-	-	-	0.60
**Adherence to Phe-free protein supplements**	0.46	0.48	**0.70**	0.64	0.67	**0.84**
**Practical impact of Phe-free protein supplements**	-	0.29	**0.82**	**0.87**	0.67	0.67
**Guilt if poor adherence to Phe-free protein supplements**	-	0.60	-	0.59	-	0.67
**Relationships within family because of Phe-free protein supplements**	-	**0.72**	-	0.69	-	0.57
**Taste – Phe-free protein supplements**	-	**0.81**	-	**0.88**	-	**0.76**
**Food temptations**	0.63	0.55	**0.77**	**0.74**	**0.78**	0.68
**Adherence to dietary protein restriction**	0.25	0.43	0.59	**0.80**	**0.81**	**0.77**
**Social impact of dietary protein restriction**	**0.74**	0.58	**0.88**	**0.86**	**0.77**	**0.83**
**Practical impact of dietary protein restriction**	-	-	**0.74**	**0.71**	**0.78**	**0.70**
**Overall impact of dietary protein restriction**	-	-	**0.74**	**0.80**	**0.88**	**0.79**
**Taste – specialty low-protein food**	-	0.47	-	0.57	-	**0.73**
**Food enjoyment**	-	0.21	-	0.31	-	**0.77**
**Guilt if dietary protein restriction not followed**	-	0.60	-	0.61	-	**0.74**
**Overall difficulty following dietary protein restriction**	-	0.55	-	0.59	-	0.54

ICC: Intraclass Correlation Coefficient; Phe: phenylalanine; PKU: phenylketonuria; QOL: quality of life. Reliability coefficients ≥0.70 are set in bold type.

**Table 8:** **Reliability coefficients of the Parent PKU-QOL questionnaire at baseline in the parent evaluable population (n = 253)**

**Parent PKU-QOL scores**	**Cronbach’s α**	**ICC**
**Child health status**	-	0.59
**Headaches**	-	0.47
**Stomach aches**	-	0.41
**Tiredness**	-	0.58
**Irritability**	-	0.65
**Aggressiveness**	-	0.69
**Moodiness**	-	0.61
**Sadness**	-	0.48
**Anxiety**	-	0.60
**Lack of concentration**	-	0.63
**Slow thinking**	-	0.60
**Emotional impact of PKU**	0.63	**0.78**
**Practical impact of PKU**	**0.74**	**0.76**
**Social impact of PKU**	**0.72**	**0.80**
**Overall impact of PKU**	**0.84**	**0.84**
**Anxiety – blood test**	**0.73**	**0.85**
**Impact of anxiety – blood test**	**0.76**	**0.78**
**Anxiety – blood Phe levels**	-	**0.79**
**Financial impact of PKU**	-	**0.78**
**Information on PKU**	-	0.63
**Adherence to Phe-free protein supplements**	-	0.42
**Management of Phe-free protein supplements**	-	0.61
**Practical impact of Phe-free protein supplements**	**0.77**	0.66
**Guilt if poor adherence to Phe-free protein supplements**	-	0.59
**Relationships within family because of Phe-free protein supplements**	-	0.59
**Adherence to dietary protein restriction**	-	0.29
**Management of dietary protein restriction**	**0.85**	**0.78**
**Practical impact of dietary protein restriction**	**0.82**	**0.79**
**Food enjoyment**	-	0.35
**Guilt if dietary protein restriction not followed**	-	**0.70**

ICC: Intraclass Correlation Coefficient; Phe: phenylalanine; PKU: phenylketonuria; QOL: quality of life. Reliability coefficients ≥0.70 are set in bold type.

#### Test-retest reliability

The analyses of the change in Child PKU-QOL scores between baseline and Week 2 for the child evaluable population showed ICCs below the threshold of acceptability for the majority of scores (0.21–0.67). The ICC exceeded the threshold for good test-retest reliability only for ‘Social impact of PKU’ (0.70), ‘Relationships within family because of supplements’ (0.72) and ‘Taste – supplements’ (0.81). See Table 7.

The ICC exceeded the threshold for good test-retest reliability for 14 of 31 Adolescent PKU-QOL scores (0.70–0.95), for 16 of 33 Adult PKU-QOL scores (0.70–0.87) and for 11 of 30 Parent PKU-QOL scores (0.70–0.85). See Tables 7 and 8.

## Discussion

The PKU-QOL questionnaires are disease specific questionnaires developed for and in collaboration with patients with PKU and parents of children with PKU to allow assessment of the impact of PKU on the HRQoL of patients. Three age-specific versions were developed for children, adolescents and adults with PKU, plus one version for parents of a child with PKU. All questionnaires were cross-culturally adapted in seven countries, by either simultaneous development (for the Netherlands, the UK, France, Germany, Italy and Spain) or proper linguistic validation (for Turkey). The impact of PKU on HRQoL being assumed to be a culturally sensitive concept, this approach was applied in an effort to optimise the cross-cultural validity of the measure. Nonetheless, even though this process is already very sophisticated and warrant a good level of cross-cultural validity of the instrument, it cannot definitely guarantee that the PKU-QOL is fully cross-culturally equivalent over all cultures (e.g. some very specific concepts in some cultures may have been missed since exploratory interviews were not conducted in all countries).

It was also decided from the beginning that the four questionnaires (child, adolescent, adult, and parent) would be similarly structured to facilitate use and comparison across all ages. While this put a constraint on the development and validation of the instrument, it is a clear strength of the questionnaire as it may further allow following patients longitudinally over time, e.g. from childhood to adulthood.

The conceptual model underlying the questionnaire was derived from the experience directly reported by the patients and parents, complemented by the opinion of healthcare professionals. The aspects to be assessed by the questionnaires appeared very clearly: PKU symptoms, impact of PKU on patients’ life and impact of the commonly used treatment options for PKU (namely dietary protein restriction and Phe-free protein supplement administration). Of note, the impact of other therapeutic interventions, in particular pharmacological treatment, did not appear as central in the experience of patients so was not included in the PKU-QOL questionnaire. This may be due to the fact that pharmacological treatment of PKU (i.e. BH4) was used in a minority of patients (e.g. it was the case of less than one fourth of patients in the validation study). Should pharmacological treatment of PKU become more frequent, an additional module might be developed for the PKU-QOL questionnaire to assess this aspect.

Hence, the novelty of the PKU-QOL questionnaires was that they have been developed for and with patients with PKU and parents of patients with PKU. This provides a strong advantage over generic HRQoL measures [19,25,26], questionnaires developed for unspecific chronic illness (e.g. Ulm Quality-of-Life Inventory for Parents of chronically ill children) [22] or even the more recent “PKU-specific” HRQoL questionnaire (PKU-QOLQ) [21], which had been adapted from a questionnaire used in another condition (juvenile diabetes) and thus had not been designed fully from the beginning for patients with PKU specifically. Because it is truly disease specific, the PKU-QOL questionnaire captures aspects that are important for PKU patients and their parents and impact their daily lives (e.g. dietary protein restriction, Phe-free protein supplement administration) that are not addressed by other HRQoL measures. Hence, the disease specific nature of the PKU-QOL allows a better acceptance of the questionnaire by patients (as demonstrated by the very good return rates and quality of completion in the validation study) and draws a more accurate picture of the impact of PKU on patients’ lives.

The measurement properties of the Adolescent, Adult and Parent PKU-QOL questionnaires were acceptable overall, although reliability of some scores (in particular single item scores) was not fully satisfactory. Measurement properties were also clearly weaker for the Child questionnaire. However, poorer results were expected for the Child questionnaire because measurement of concepts as complex as HRQoL is known to be challenging for children to self-report [43]. The clinical validity of the PKU-QOL confirmed the hypothesis that more compromised HRQoL is found in patients with more severe classical PKU, and exhibiting worse health status. These findings tend to show that the PKU-QOL scores seem to capture the difference in the experience of patients with the management of their disease (diet and Phe-free protein supplement), as patients with classical PKU have a more strict management in terms of dietary restriction and Phe-free protein supplement intakes. Further analysis of PKU-QOL scores and comparisons according to PKU severity, treatment with pharmacological adjunctive treatment (e.g. BH4) and health status as assessed by a clinician is presented in details elsewhere [34].

A remarkable feature of the validation study of the PKU-QOL was that there were about as many males as females in the child and adolescent patient samples while there were substantially more women in both the adult and parent samples. This may reflect the higher number of female PKU patients who continue genetics care as adults, in particular due to the risks associated with pregnancy in PKU, and the persisting central role of mothers in the management of children.

A skewed distribution of responses was observed for most of PKU-QOL items. This finding was not unexpected given the overall good health status of the population of patients with PKU and limited impact of PKU or its treatment on the lives of many patients, which were reported previously and confirmed at all stages of our research. While this is not an issue in terms of measurement by the PKU-QOL questionnaire (since it reflects the reality of patients’ experience), from an analytical point of view, this may affect the estimation of correlation coefficients on items (e.g. in the multi-trait analysis) and even on scores (e.g. in the concurrent validity analysis): This could have weakened the convergent validity of results and could explain why the correlation between PKU-QOL scores and generic HRQoL measures could be regarded at best as moderate.

The validation study of the PKU-QOL had some features that potentially affected the results. First, even if this study cohort was large for a disease as rare as PKU (34 sites in seven countries), the samples available for the finalisation and validation of each version of the PKU-QOL questionnaire were relatively small for this kind of exercise, which generally requires at least 200 patients. However, these features were anticipated and addressed by using simple statistical methods adapted to small samples (such as rank order correlations). Second, the severity of PKU was only characterised in the study using Phe level at diagnosis. This decision was made in an effort to keep the study as simple as possible with the minimum burden on the clinicians, but another indicator of severity of PKU, Phe tolerance, might have allowed complementary analyses to be performed. Third, the study was conducted in seven countries with clearly different cultures. This should be considered as a strength of the PKU-QOL questionnaires as they have now been validated in diverse cultural settings. However, the cultural differences may also introduce some heterogeneity, adding on to the variability of the results, and potentially increasing the risk of loss of robustness of the data.

Future applications of these questionnaires include the possibility of further targeted evaluation of HRQoL as impacted by PKU throughout the lifespan. This would allow better understanding and documentation of the implications of traditional dietary therapy requirements, pharmacological treatment (e.g. BH4 supplementation) and characteristic psychological issues impacting HRQoL. These questionnaires allow prospective observations of HRQoL over time and evaluation of evolution of patients’ perceptions with age, as well as the assessment of differences between parents’ and patients’ perceptions. In addition, the availability of disease specific PKU-QOL questionnaires in seven languages will facilitate their comparative use across population included in international clinical trials, increase knowledge of the impact of PKU on the HRQoL of patients and parents in different countries, and allow exploring HRQoL cross-cultural differences in PKU patients and their parents. The PKU-QOL questionnaires may also help to monitor the efficacy of therapeutic and non-therapeutic (e.g. nutrition, psychotherapeutic consulting) treatment strategies by assessing their impact on HRQoL. Finally, clinical use of this questionnaire will help to address gaps in understanding between physicians, patients and parents concerning their perceptions of HRQoL as affected by PKU.

A better understanding of the impact of PKU on patients and their families still requires further research. To this end, we invite researchers to use the PKU-QOL questionnaires, which are the first validated questionnaires specifically developed for this purpose. Further data collection would create an additional body of evidence that will allow better understanding for patients, parents and physicians. This, in turn may allow improved quality and consistency of care in this chronic disease.

## Conclusions

Our study aimed to develop and validate a disease specific PKU HRQoL questionnaire – the first self-administered questionnaire designed to comprehensively assess the impact of PKU and its treatment on the HRQoL of patients and their parents. The questionnaires were developed in seven languages, in four different populations: children aged 9–11 years (Child PKU-QOL), adolescents aged 12–17 years (Adolescent PKU-QOL), adults aged 18 years and above (Adult PKU-QOL), and parents of patients with PKU (Parent PKU-QOL). The four questionnaires assess comprehensively the different factors of life specific to PKU and the treatment required (such as symptoms and feelings, daily life, administration of Phe-free protein supplements, and dietary protein restriction), and share a very similar structure, but still reflect the specific realities of each of the populations. A comprehensive methodology was applied to validate the questionnaires in a prospective validation study demonstrating that all questionnaires had satisfactory measurement properties and can be used for evaluation of HRQoL in PKU patients and their parents. The PKU-QOL questionnaires will allow assessing and documenting how patients’ perceptions evolve according to age, increasing our knowledge of the impact of PKU on patients and parents’ life in different countries, and eventually helping monitor the efficacy of therapeutic strategies.

### Intellectual property and condition of use

The PKU-QOL questionnaires are protected by international copyright – PKU-QOL © Merck Serono S.A. - Geneva – 2010-.

The PKU-QOL questionnaire is available freely for use in individual medical practice and in non-funded academic research. Access to the questionnaire, as well as further information on, or permission to use the PKU-QOL and/or its translations, can be found on http://www.proqolid.org.

## Additional files

Additional file 1:
**Multitrait analysis of the Child PKU-QOL.**
Additional file 2:
**Clinical Validity of the Child PKU-QOL.**
Additional file 3:
**Clinical Validity of the Adolescent PKU-QOL.**
Additional file 4:
**Clinical Validity of the Adult PKU-QOL.**
Additional file 5:
**Clinical Validity of the Parent PKU-QOL.**

## Abbreviations

ANOVA: Analysis of variance
BH_4_: Tetrahydrobiopterin
CHQ-PF: Child Health Questionnaire–Parent Form
HRQoL: Health-related quality of life
ICC: Intraclass correlation coefficient
PAH: Phenylalanine hydroxylase
PedsQL: Pediatric Quality-of-Life Inventory
Phe: Phenylalanine
PKU: Phenylketonuria
PKU-QOLQ: PKU-specific self-report QOL questionnaire
QOL: Quality of life
SF-36: 36-item Short Form
