# Supplementary material for: Development and psychometric validation of measures to assess the impact of phenylketonuria and its dietary treatment on patients’ and parents’ quality of life: the phenylketonuria – quality of life (PKU-QOL) questionnaires
Source: Orphanet J Rare Dis. 2015 May 10;10:59. doi: 10.1186/s13023-015-0261-6 (PMC4449597; doi:10.1186/s13023-015-0261-6)
Supplement: Additional file 1: — Multitrait analysis of the Child PKU-QOL. [file 13023_2015_261_MOESM1_ESM.pdf]

Additional file 1

Multitrait analysis of the Child PKU-QOL

Table 1-1 - Item convergent and discriminant criteria of the in 'PKU in general' module in the child evaluable population (n=92)

Domains	# items	Range of item-scale correlations	% of items meeting convergent criterion[a]	% of items meeting discriminant criterion[b]
Emotional impact of PKU	3	0.16–0.40	33%	67%
Practical impact of PKU	2	0.32–0.32	0%	50%
Social impact of PKU	3	0.35–0.41	33%	67%
Anxiety – blood test	2	0.25–0.25	0%	100%

PKU: phenylketonuria.

[a]Item Convergent criterion met if item-scale correlation greater than 0.4.

[b]Item Discriminant validity criterion met if the correlation of an item with its own scale is greater than the correlation of this item with all the other scales.

Table 1-2 - Item convergent and discriminant criteria of 'Supplement administration' module in the child evaluable population (n=92)

Domains	# items	Range of item-scale correlations	% of items meeting convergent criterion[a]	% of items meeting discriminant criterion[b]
Adherence to supplements	2	0.23–0.23	0%	50%
Social impact of supplements	2	0.29–0.29	0%	50%

[a]Item Convergent criterion met if item-scale correlation greater than 0.4.

[b]Item Discriminant validity criterion met if the correlation of an item with its own scale is greater than the correlation of this item with all the other scales.

.

Table 1-3 - Item convergent and discriminant criteria of the 'Dietary protein restriction' module in the child evaluable population (n=92)

Domains	# items	Range of item-scale correlations	% of items meeting convergent criterion[a]	% of items meeting discriminant criterion[b]
Food temptations	2	0.47, 0.47	100%	0%
Adherence to dietary protein restriction	2	0.30, 0.30	0%	50%
Social impact of dietary protein restriction	5	0.31, 0.65	80%	60%

[a]Item Convergent criterion met if item-scale correlation greater than 0.4.

[b]Item Discriminant validity criterion met if the correlation of an item with its own scale is greater than the correlation of this item with all the other scales..

Multitrait analysis of the Ado PKU-QOL questionnaire

Table 1-4 - Item convergent and discriminant criteria of the 'PKU in General' module in the adolescent evaluable population (n=110)

Domains	# items	Range of item-scale correlations	% of items meeting convergent criterion[a]	% of items meeting discriminant criterion[b]
Emotional impact of PKU	5	0.41, 0.60	100%	60%
Practical impact of PKU	3	0.24, 0.52	33%	0%
Social impact of PKU	3	–0.11, 0.26	0%	33%
Anxiety – blood test	2	0.60, 0.60	100%	100%

PKU: phenylketonuria.

[a]Item Convergent criterion met if item-scale correlation greater than 0.4.

[b]Item Discriminant validity criterion met if the correlation of an item with its own scale is greater than the correlation of this item with all the other scales.

Table 1-5 - Item convergent and discriminant criteria of the 'Supplement administration' module in the adolescent evaluable population (n=110)

Domains	# items	Range of item-scale correlations	% of items meeting convergent criterion[a]	% of items meeting discriminant criterion[b]
Adherence to supplements	4	0.40–0.60	75%	100%
Practical impact of supplements	4	0.56–0.65	100%	100%

[a]Item Convergent criterion met if item-scale correlation greater than 0.4.

[b]Item Discriminant validity criterion met if the correlation of an item with its own scale is greater than the correlation of this item with all the other scales.

Table 1-6 - Item convergent and discriminant criteria of the 'Dietary protein restriction' module in the adolescent evaluable population (n=110)

Domains	# items	Range of item-scale correlations	% of items meeting convergent criterion[a]	% of items meeting discriminant criterion[b]
Food temptations	2	0.58–0.58	100%	100%
Adherence to dietary protein restriction	4	0.30–0.46	75%	50%
Practical impact of dietary protein restriction	7	0.12–0.59	57%	57%
Social impact of dietary protein restriction	5	0.51–0.86	100%	100%

[a]Item Convergent criterion met if item-scale correlation greater than 0.4.

[b]Item Discriminant validity criterion met if the correlation of an item with its own scale is greater than the correlation of this item with all the other scales.

Multitrait analysis of the Adult PKU-QOL questionnaire

Table 1-7 - Item convergent and discriminant criteria of the 'PKU in general' module in the adult evaluable population (n=104)

**Domains**	**# items**	**Range of item-scale correlations**	**% of items meeting convergent criterion[a]**	**% of items meeting discriminant criterion[b]**
Emotional impact of PKU	5	0.42–0.58	100%	40%
Practical impact of PKU	4	0.31–0.39	0%	75%
Social impact of PKU	4	0.37–0.46	75%	75%
Anxiety – blood test	2	0.41–0.41	100%	50%

PKU: phenylketonuria.

[a]Item Convergent criterion met if item-scale correlation greater than 0.4.

[b]Item Discriminant validity criterion met if the correlation of an item with its own scale is greater than the correlation of this item with all the other scales.

Table 1-8 - Item convergent and discriminant criteria of the 'Supplement administration' module in the adult evaluable population (n=104)

**Domains**	**# items**	**Range of item-scale correlations**	**% of items meeting convergent criterion[a]**	**% of items meeting discriminant criterion[b]**
Adherence to supplements	3	0.47–0.57	100%	100%
Practical impact of supplements	4	0.33–0.55	75%	75%

[a]Item Convergent criterion met if item-scale correlation greater than 0.4.

[b]Item Discriminant validity criterion met if the correlation of an item with its own scale is greater than the correlation of this item with all the other scales.

Table 1-9 - Item convergent and discriminant criteria of the 'Dietary protein restriction' module in the adult evaluable population (n=104)

**Domains**	**# items**	**Range of item-scale correlations**	**% of items meeting convergent criterion[a]**	**% of items meeting discriminant criterion[b]**
Food temptations	2	0.67–0.67	100%	100%
Adherence to dietary protein restriction	5	0.53–0.80	100%	100%
Practical impact of dietary protein restriction	7	0.31–0.66	86%	57%
Social impact of dietary protein restriction	6	0.42–0.65	100%	67%

[a]Item Convergent criterion met if item-scale correlation greater than 0.4.

[b]Item Discriminant validity criterion met if the correlation of an item with its own scale is greater than the correlation of this item with all the other scales.

Multitrait analysis of the Parent PKU-QOL questionnaire

Table 1-10 - Item convergent and discriminant criteria of the 'PKU in general' module in the parent evaluable population (n=253)

Domains	# items	Range of item-scale correlations	% of items meeting convergent criterion[a]	% of items meeting discriminant criterion[b]
Emotional impact of PKU	4	0.34–0.54	50%	50%
Practical impact of PKU	6	0.25–0.59	83%	83%
Social impact of PKU	5	0.31–0.49	80%	60%
Anxiety – blood test	2	0.54–0.54	100%	0%
Impact of anxiety – blood test	2	0.64–0.64	100%	0%

PKU: phenylketonuria.

[a]Item Convergent criterion met if item-scale correlation greater than 0.4.

[b]Item Discriminant validity criterion met if the correlation of an item with its own scale is greater than the correlation of this item with all the other scales.

Table 1-11 - Item convergent and discriminant criteria of 'Dietary protein restriction' module in the parent evaluable population (n=253)

Domains	# items	Range of item-scale correlations	% of items meeting convergent criterion[a]	% of items meeting discriminant criterion[b]
Practical impact of dietary protein restriction	7	0.47–0.68	100%	100%
Management of dietary protein restriction	6	0.56–0.68	100%	100%

[a]Item Convergent criterion met if item-scale correlation greater than 0.4.

[b]Item Discriminant validity criterion met if the correlation of an item with its own scale is greater than the correlation of this item with all the other scales.
