# Supplementary material for: Development and psychometric validation of measures to assess the impact of phenylketonuria and its dietary treatment on patients’ and parents’ quality of life: the phenylketonuria – quality of life (PKU-QOL) questionnaires
Source: Orphanet J Rare Dis. 2015 May 10;10:59. doi: 10.1186/s13023-015-0261-6 (PMC4449597; doi:10.1186/s13023-015-0261-6)
Supplement: Additional file 2: — Clinical Validity of the Child PKU-QOL. [file 13023_2015_261_MOESM2_ESM.pdf]

Additional file 2

Clinical validity of the Child PKU-QOL questionnaire

Table 2-1 - Clinical validity of the Child PKU-QOL Questionnaire: comparison of PKU-QOL scores according to patient overall health status as rated by the clinician in the child evaluable population (n=92)

Scores of 0 indicate no impact (or no symptom for symptom scores), scores of 25 indicate a mild impact (or rare symptoms), scores of 50 indicate a moderate impact (or occasional symptoms), scores of 75 indicate major impact (or frequent symptom), scores of 100 indicate extremely severe impact (or very frequent symptoms)

		Overall assessment of health status by the clinician[a]				
		**Fair (n=2)**	**Good (n=19)**	**Very good (n=41)**	**Excellent (n=28)**	**p-value (Kruskal-Wallis)**
**Headaches**	n (missing)	2 (0)	18 (1)	40 (1)	26 (2)	0.8285
	Median (Q1–Q3)	12.5 (0.0–25.0)	25.0 (0.0–25.0)	0.0 (0.0–25.0)	0.0 (0.0–50.0)	
**Stomach aches**	n (missing)	2 (0)	18 (1)	40 (1)	26 (2)	0.5176
	Median (Q1–Q3)	50.0 (0.0–100.0)	0.0 (0.0–25.0)	0.0 (0.0–25.0)	0.0 (0.0–25.0)	
**Tiredness**	n (missing)	2 (0)	18 (1)	40 (1)	26 (2)	0.3815
	Median (Q1–Q3)	62.5 (25.0–100.0)	50.0 (25.0–50.0)	25.0 (0.0–50.0)	50.0 (25.0–50.0)	
**Irritability**	n (missing)	2 (0)	18 (1)	40 (1)	26 (2)	0.7379
	Median (Q1–Q3)	50.0 (0.0–100.0)	0.0 (0.0–25.0)	0.0 (0.0–50.0)	0.0 (0.0–25.0)	
**Aggressiveness**	n (missing)	2 (0)	18 (1)	40 (1)	26 (2)	0.5338
	Median (Q1–Q3)	50.0 (0.0–100.0)	0.0 (0.0–25.0)	0.0 (0.0–25.0)	0.0 (0.0–0.0)	
**Moodiness**	n (missing)	2 (0)	18 (1)	40 (1)	26 (2)	0.2045
	Median (Q1–Q3)	50.0 (0.0–100.0)	0.0 (0.0–25.0)	12.5 (0.0–50.0)	0.0 (0.0–25.0)	
**Sadness**	n (missing)	2 (0)	18 (1)	40 (1)	27 (1)	0.1795
	Median (Q1–Q3)	62.5 (25.0–100.0)	0.0 (0.0–25.0)	0.0 (0.0–25.0)	0.0 (0.0–25.0)	
**Anxiety**	n (missing)	2 (0)	18 (1)	40 (1)	26 (2)	0.3472
	Median (Q1–Q3)	25.0 (0.0–50.0)	0.0 (0.0–0.0)	0.0 (0.0–25.0)	0.0 (0.0–0.0)	
**Lack of concentration**	n (missing)	2 (0)	17 (2)	34 (7)	25 (3)	0.5387
	Median (Q1–Q3)	50.0 (25.0–75.0)	25.0 (0.0–25.0)	0.0 (0.0–50.0)	0.0 (0.0–25.0)	
**Slow thinking**	n (missing)	2 (0)	17 (2)	33 (8)	25 (3)	0.4529
	Median (Q1–Q3)	37.5 (25.0–50.0)	25.0 (0.0–50.0)	0.0 (0.0–50.0)	25.0 (0.0–25.0)	
**Emotional impact of PKU**	n (missing)	2 (0)	18 (1)	40 (1)	26 (2)	0.6903
	Median (Q1–Q3)	41.7 (25.0–58.3)	37.5 (16.7–58.3)	29.2 (12.5–54.2)	29.2 (16.7–41.7)	
**Practical impact of PKU**	n (missing)	2 (0)	18 (1)	40 (1)	27 (1)	0.9002
	Median (Q1–Q3)	25.0 (0.0–50.0)	0.0 (0.0–12.5)	0.0 (0.0–25.0)	0.0 (0.0–25.0)	

		Overall assessment of health status by the clinician[a]				
		**Fair (n=2)**	**Good (n=19)**	**Very good (n=41)**	**Excellent (n=28)**	**p-value (Kruskal-Wallis)**
**Social impact of PKU**	n (missing)	2 (0)	18 (1)	40 (1)	26 (2)	0.0856
	Median (Q1–Q3)	37.5 (16.7–58.3)	25.0 (16.7–33.3)	16.7 (8.3–25.0)	8.3 (0.0–16.7)	
**Overall impact of PKU**	n (missing)	2 (0)	18 (1)	40 (1)	26 (2)	0.1611
	Median (Q1–Q3)	35.9 (28.1–43.8)	28.1 (18.8–34.4)	18.8 (12.5–29.7)	17.2 (9.4–25.0)	
**Anxiety – blood test**	n (missing)	2 (0)	17 (2)	40 (1)	27 (1)	0.2659
	Median (Q1–Q3)	0.0 (0.0–0.0)	0.0 (0.0–12.5)	12.5 (0.0–25.0)	0.0 (0.0–12.5)	
**Anxiety – Phe levels**	n (missing)	2 (0)	18 (1)	40 (1)	27 (1)	0.9023
	Median (Q1–Q3)	50.0 (25.0–75.0)	25.0 (25.0–50.0)	50.0 (25.0–87.5)	50.0 (0.0–75.0)	
**Adherence to supplements**	n (missing)	2 (0)	17 (2)	39 (2)	24 (4)	0.8713
	Median (Q1–Q3)	50.0 (0.0–100.0)	0.0 (0.0–12.5)	0.0 (0.0–12.5)	0.0 (0.0–12.5)	
**Practical impact of supplements**	n (missing)	2 (0)	17 (2)	39 (2)	25 (3)	0.5710
	Median (Q1–Q3)	50.0 (0.0–100.0)	0.0 (0.0–75.0)	0.0 (0.0–25.0)	0.0 (0.0–0.0)	
**Guilt if poor adherence to supplements**	n (missing)	2 (0)	17 (2)	38 (3)	23 (5)	0.4441
	Median (Q1–Q3)	12.5 (0.0–25.0)	25.0 (25.0–50.0)	50.0 (25.0–100.0)	25.0 (0.0–75.0)	
**Relationships within family because of supplements**	n (missing)	2 (0)	17 (2)	39 (2)	25 (3)	0.3638
	Median (Q1–Q3)	25.0 (0.0–50.0)	0.0 (0.0–0.0)	0.0 (0.0–25.0)	0.0 (0.0–0.0)	
**Taste – supplements**	n (missing)	2 (0)	17 (2)	39 (2)	23 (5)	0.0629
	Median (Q1–Q3)	0.0 (0.0–0.0)	25.0 (0.0–50.0)	25.0 (0.0–50.0)	50.0 (25.0–75.0)	
**Food temptations**	n (missing)	2 (0)	16 (3)	39 (2)	26 (2)	0.5450
	Median (Q1–Q3)	31.3 (25.0–37.5)	18.8 (0.0–25.0)	25.0 (0.0–50.0)	31.3 (0.0–50.0)	
**Adherence to dietary protein-restriction**	n (missing)	2 (0)	16 (3)	38 (3)	27 (1)	0.0589
	Median (Q1–Q3)	37.5 (25.0–50.0)	0.0 (0.0–12.5)	0.0 (0.0–12.5)	0.0 (0.0–0.0)	
**Social impact of dietary protein restriction**	n (missing)	2 (0)	16 (3)	39 (2)	27 (1)	0.8839
	Median (Q1–Q3)	22.5 (5.0–40.0)	17.5 (0.0–35.0)	15.0 (5.0–25.0)	15.0 (0.0–30.0)	
**Taste – low-protein food**	n (missing)	2 (0)	16 (3)	37 (4)	22 (6)	0.3896
	Median (Q1–Q3)	0.0 (0.0–0.0)	12.5 (0.0–25.0)	25.0 (0.0–25.0)	0.0 (0.0–25.0)	
**Food enjoyment**	n (missing)	2 (0)	18 (1)	40 (1)	27 (1)	0.2676
	Median (Q1–Q3)	50.0 (0.0–100.0)	0.0 (0.0–25.0)	25.0 (0.0–25.0)	0.0 (0.0–25.0)	
**Guilt if dietary protein restriction not followed**	n (missing)	2 (0)	16 (3)	38 (3)	25 (3)	0.8701
	Median (Q1–Q3)	62.5 (25.0–100.0)	62.5 (25.0–87.5)	50.0 (25.0–100.0)	50.0 (0.0–100.0)	
**Overall difficulty following dietary protein restriction**	n (missing)	2 (0)	16 (3)	39 (2)	27 (1)	0.3617
	Median (Q1–Q3)	0.0 (0.0–0.0)	12.5 (0.0–25.0)	25.0 (0.0–25.0)	0.0 (0.0–25.0)	

		Overall assessment of health status by the clinician[a]				
		**Fair (n=2)**	**Good (n=19)**	**Very good (n=41)**	**Excellent (n=28)**	**p-value (Kruskal-Wallis)**

Phe: phenylalanine; PKU: phenylketonuria; Q: quartile; QOL: quality of life.
[a]Modality Poor (n=0).

Table 2-2 - Clinical validity of the Child PKU-QOL questionnaire: comparison of PKU-QOL scores according to severity of PKU defined by Phe levels at diagnosis in the child evaluable population (n=92)

Scores of 0 indicate no impact (or no symptom for symptom scores), scores of 25 indicate a mild impact (or rare symptoms), scores of 50 indicate a moderate impact (or occasional symptoms), scores of 75 indicate major impact (or frequent symptom), scores of 100 indicate extremely severe impact (or very frequent symptoms)

		Severity of PKU		
		**Mild-moderate PKU (Phe level: 600–1200 µmol/L) (n=22)**	**Classic PKU (Phe level: >1200 µmol/L) (n=66)**	**p-value (Mann–Whitney /Wilcoxon)**
**Headaches**	n (missing)	20 (2)	64 (2)	0.8170
	Median (Q1–Q3)	0.0 (0.0–37.5)	0.0 (0.0–25.0)	
**Stomach aches**	n (missing)	20 (2)	64 (2)	0.4553
	Median (Q1–Q3)	0.0 (0.0–50.0)	0.0 (0.0–25.0)	
**Tiredness**	n (missing)	20 (2)	64 (2)	0.6657
	Median (Q1–Q3)	50.0 (0.0–62.5)	25.0 (25.0–50.0)	
**Irritability**	n (missing)	20 (2)	64 (2)	0.9479
	Median (Q1–Q3)	0.0 (0.0–50.0)	0.0 (0.0–25.0)	
**Aggressiveness**	n (missing)	20 (2)	64 (2)	0.2383
	Median (Q1–Q3)	0.0 (0.0–50.0)	0.0 (0.0–12.5)	
**Moodiness**	n (missing)	20 (2)	64 (2)	0.3343
	Median (Q1–Q3)	0.0 (0.0–50.0)	0.0 (0.0–25.0)	
**Sadness**	n (missing)	21 (1)	64 (2)	0.4173
	Median (Q1–Q3)	0.0 (0.0–50.0)	0.0 (0.0–25.0)	
**Anxiety**	n (missing)	20 (2)	64 (2)	0.7245
	Median (Q1–Q3)	0.0 (0.0–0.0)	0.0 (0.0–12.5)	
**Lack of concentration**	n (missing)	19 (3)	57 (9)	0.8866
	Median (Q1–Q3)	0.0 (0.0–50.0)	25.0 (0.0–25.0)	
**Slow thinking**	n (missing)	19 (3)	56 (10)	0.9379
	Median (Q1–Q3)	25.0 (0.0–25.0)	25.0 (0.0–50.0)	
**Emotional impact of PKU**	n (missing)	21 (1)	63 (3)	0.4957
	Median (Q1–Q3)	33.3 (16.7–50.0)	25.0 (16.7–50.0)	
**Practical impact of PKU**	n (missing)	21 (1)	64 (2)	0.3251
	Median (Q1–Q3)	0.0 (0.0–25.0)	0.0 (0.0–12.5)	
**Social impact of PKU**	n (missing)	21 (1)	63 (3)	0.8296
	Median (Q1–Q3)	16.7 (0.0–33.3)	16.7 (8.3–25.0)	
**Overall impact of PKU**	n (missing)	21 (1)	63 (3)	[illegible]

		Severity of PKU		
		**Mild-moderate PKU (Phe level: 600–1200 µmol/L) (n=22)**	**Classic PKU (Phe level: >1200 µmol/L) (n=66)**	**p-value (Mann–Whitney /Wilcoxon)**
	Median (Q1–Q3)	18.8 (12.5–37.5)	18.8 (9.4–31.3)	
**Anxiety – blood test**	n (missing)	21 (1)	63 (3)	0.1812
	Median (Q1–Q3)	0.0 (0.0–12.5)	12.5 (0.0–25.0)	
**Anxiety – Phe levels**	n (missing)	21 (1)	64 (2)	0.1019
	Median (Q1–Q3)	50.0 (25.0–100.0)	25.0 (12.5–75.0)	
**Adherence to supplements**	n (missing)	17 (5)	63 (3)	0.3270
	Median (Q1–Q3)	12.5 (0.0–25.0)	0.0 (0.0–12.5)	
**Practical impact of supplements**	n (missing)	18 (4)	63 (3)	0.3909
	Median (Q1–Q3)	0.0 (0.0–0.0)	0.0 (0.0–50.0)	
**Guilt if poor adherence to supplements**	n (missing)	17 (5)	61 (5)	0.0089
	Median (Q1–Q3)	0.0 (0.0–50.0)	50.0 (25.0–100.0)	
**Relationships within family because of supplements**	n (missing)	18 (4)	63 (3)	0.0903
	Median (Q1–Q3)	0.0 (0.0–50.0)	0.0 (0.0–0.0)	
**Taste – supplements**	n (missing)	17 (5)	62 (4)	0.0267
	Median (Q1–Q3)	50.0 (50.0–50.0)	25.0 (0.0–50.0)	
**Food temptations**	n (missing)	19 (3)	63 (3)	0.1289
	Median (Q1–Q3)	37.5 (0.0–75.0)	25.0 (0.0–50.0)	
**Adherence to dietary protein restriction**	n (missing)	19 (3)	62 (4)	0.7607
	Median (Q1–Q3)	0.0 (0.0–12.5)	0.0 (0.0–12.5)	
**Social impact of dietary protein restriction**	n (missing)	19 (3)	63 (3)	0.1962
	Median (Q1–Q3)	15.0 (5.0–45.0)	12.5 (5.0–30.0)	
**Taste – low-protein food**	n (missing)	13 (9)	62 (4)	0.7134
	Median (Q1–Q3)	25.0 (0.0–25.0)	25.0 (0.0–25.0)	
**Food enjoyment**	n (missing)	21 (1)	64 (2)	0.7867
	Median (Q1–Q3)	0.0 (0.0–50.0)	0.0 (0.0–25.0)	
**Guilt if dietary protein restriction not followed**	n (missing)	19 (3)	60 (6)	0.0592
	Median (Q1–Q3)	0.0 (0.0–100.0)	62.5 (25.0–100.0)	
**Overall difficulty following dietary protein restriction**	n (missing)	19 (3)	63 (3)	0.6642
	Median (Q1–Q3)	0.0 (0.0–50.0)	0.0 (0.0–25.0)	

Phe: phenylalanine; PKU: phenylketonuria; Q: quartile; QOL: quality of life.
