# Supplementary material for: Development and psychometric validation of measures to assess the impact of phenylketonuria and its dietary treatment on patients’ and parents’ quality of life: the phenylketonuria – quality of life (PKU-QOL) questionnaires
Source: Orphanet J Rare Dis. 2015 May 10;10:59. doi: 10.1186/s13023-015-0261-6 (PMC4449597; doi:10.1186/s13023-015-0261-6)
Supplement: Additional file 3: — Clinical Validity of the Adolescent PKU-QOL. [file 13023_2015_261_MOESM3_ESM.pdf]

Additional file 3

Clinical validity of the Adolescent PKU-QOL questionnaire

Table 3-1 - Clinical validity of the Adolescent PKU-QOL questionnaire: comparison of PKU-QOL scores according to patient overall health status as rated by the clinician in the adolescent evaluable population (n=110)

Scores of 0 indicate no impact (or no symptom for symptom scores), scores of 25 indicate a mild impact (or rare symptoms), scores of 50 indicate a moderate impact (or occasional symptoms), scores of 75 indicate major impact (or frequent symptom), scores of 100 indicate extremely severe impact (or very frequent symptoms)

		Overall assessment of health status by the clinician[a]			
		**Good (n=38)**	**Very good (n=42)**	**Excellent (n=30)**	**p-value (Kruskal-Wallis)**
**Self-rated health status**	n (missing)	35 (3)	40 (2)	30 (0)	0.0012
	Median (Q1–Q3)	50.0 (25.0–50.0)	50.0 (25.0–50.0)	12.5 (0.0–50.0)	
**Headaches**	n (missing)	35 (3)	40 (2)	30 (0)	0.9077
	Median (Q1–Q3)	0.0 (0.0–50.0)	12.5 (0.0–37.5)	12.5 (0.0–25.0)	
**Stomach aches**	n (missing)	35 (3)	40 (2)	30 (0)	0.3551
	Median (Q1–Q3)	0.0 (0.0–25.0)	0.0 (0.0–25.0)	0.0 (0.0–25.0)	
**Tiredness**	n (missing)	35 (3)	40 (2)	30 (0)	0.2638
	Median (Q1–Q3)	50.0 (25.0–75.0)	50.0 (25.0–50.0)	25.0 (25.0–50.0)	
**Lack of concentration**	n (missing)	35 (3)	42 (0)	30 (0)	0.0566
	Median (Q1–Q3)	25.0 (0.0–50.0)	0.0 (0.0–25.0)	0.0 (0.0–25.0)	
**Slow thinking**	n (missing)	35 (3)	42 (0)	30 (0)	0.5488
	Median (Q1–Q3)	0.0 (0.0–25.0)	0.0 (0.0–25.0)	0.0 (0.0–25.0)	
**Irritability**	n (missing)	35 (3)	42 (0)	30 (0)	0.0618
	Median (Q1–Q3)	50.0 (0.0–50.0)	0.0 (0.0–25.0)	25.0 (0.0–25.0)	
**Aggressiveness**	n (missing)	35 (3)	42 (0)	30 (0)	0.3053
	Median (Q1–Q3)	0.0 (0.0–50.0)	0.0 (0.0–0.0)	0.0 (0.0–0.0)	
**Moodiness**	n (missing)	35 (3)	42 (0)	30 (0)	0.3366
	Median (Q1–Q3)	25.0 (0.0–25.0)	0.0 (0.0–25.0)	12.5 (0.0–50.0)	
**Sadness**	n (missing)	35 (3)	42 (0)	30 (0)	0.5701
	Median (Q1–Q3)	0.0 (0.0–25.0)	0.0 (0.0–25.0)	0.0 (0.0–25.0)	
**Anxiety**	n (missing)	35 (3)	42 (0)	30 (0)	0.0770
	Median (Q1–Q3)	0.0 (0.0–25.0)	0.0 (0.0–25.0)	0.0 (0.0–0.0)	
**Emotional impact of PKU**	n (missing)	35 (3)	41 (1)	30 (0)	0.1650
	Median (Q1–Q3)	30.0 (25.0–55.0)	30.0 (15.0–40.0)	30.0 (20.0–35.0)	
**Practical impact of**	n (missing)	16 (22)	25 (17)	11 (19)	0.0950

		Overall assessment of health status by the clinician[a]			
		**Good (n=38)**	**Very good (n=42)**	**Excellent (n=30)**	**p-value (Kruskal-Wallis)**
**PKU**	Median (Q1–Q3)	16.7 (4.2–33.3)	8.3 (0.0–8.3)	8.3 (0.0–8.3)	
**Social impact of PKU**	n (missing)	35 (3)	41 (1)	29 (1)	0.7230
	Median (Q1–Q3)	16.7 (8.3–25.0)	8.3 (8.3–25.0)	8.3 (8.3–25.0)	
**Overall impact of PKU**	n (missing)	35 (3)	41 (1)	30 (0)	0.0931
	Median (Q1–Q3)	25.0 (15.0–38.9)	20.0 (9.1–27.3)	17.5 (12.5–25.0)	
**Anxiety – blood test**	n (missing)	35 (3)	41 (1)	30 (0)	0.9904
	Median (Q1–Q3)	0.0 (0.0–12.5)	0.0 (0.0–12.5)	0.0 (0.0–12.5)	
**Anxiety – Phe levels**	n (missing)	35 (3)	41 (1)	30 (0)	0.5299
	Median (Q1–Q3)	50.0 (0.0–75.0)	25.0 (0.0–50.0)	25.0 (0.0–50.0)	
**Adherence to supplements**	n (missing)	31 (7)	37 (5)	24 (6)	0.2385
	Median (Q1–Q3)	8.3 (0.0–33.3)	6.3 (0.0–25.0)	6.3 (0.0–17.7)	
**Practical impact of supplements**	n (missing)	32 (6)	37 (5)	25 (5)	0.4469
	Median (Q1–Q3)	12.5 (0.0–34.4)	12.5 (0.0–25.0)	6.3 (0.0–31.3)	
**Guilt if poor adherence to supplements**	n (missing)	31 (7)	37 (5)	26 (4)	0.8585
	Median (Q1–Q3)	25.0 (25.0–75.0)	25.0 (0.0–75.0)	50.0 (25.0–75.0)	
**Relationships within family because of supplements**	n (missing)	32 (6)	37 (5)	25 (5)	0.9829
	Median (Q1–Q3)	0.0 (0.0–25.0)	0.0 (0.0–25.0)	0.0 (0.0–25.0)	
**Taste – supplements**	n (missing)	32 (6)	37 (5)	25 (5)	0.6335
	Median (Q1–Q3)	50.0 (25.0–50.0)	50.0 (25.0–50.0)	50.0 (25.0–50.0)	
**Food temptations**	n (missing)	30 (8)	38 (4)	23 (7)	0.3603
	Median (Q1–Q3)	25.0 (0.0–50.0)	25.0 (0.0–50.0)	12.5 (0.0–37.5)	
**Adherence to dietary protein restriction**	n (missing)	29 (9)	38 (4)	22 (8)	0.0826
	Median (Q1–Q3)	12.5 (0.0–33.3)	8.3 (0.0–18.8)	0.0 (0.0–12.5)	
**Social impact of dietary protein restriction**	n (missing)	32 (6)	38 (4)	23 (7)	0.3555
	Median (Q1–Q3)	7.5 (0.0–37.5)	5.0 (0.0–10.0)	5.0 (0.0–20.0)	
**Practical impact of dietary protein restriction**	n (missing)	26 (12)	26 (16)	18 (12)	0.6588
	Median (Q1–Q3)	26.8 (17.9–42.9)	24.3 (14.3–30.0)	28.6 (20.0–30.0)	
**Overall impact of dietary protein restriction**	n (missing)	30 (8)	36 (6)	22 (8)	0.1773
	Median (Q1–Q3)	19.4 (11.4–36.4)	13.1 (5.7–23.9)	18.2 (9.4–22.5)	
**Taste – low-protein food**	n (missing)	31 (7)	34 (8)	19 (11)	0.2281
	Median (Q1–Q3)	25.0 (0.0–50.0)	25.0 (0.0–25.0)	25.0 (0.0–50.0)	
**Food enjoyment**	n (missing)	30 (8)	37 (5)	23 (7)	0.4605
	Median (Q1–Q3)	0.0 (0.0–25.0)	0.0 (0.0–25.0)	0.0 (0.0–25.0)	
**Guilt if dietary protein restriction not followed**	n (missing)	31 (7)	39 (3)	23 (7)	0.6572
	Median (Q1–Q3)	50.0 (0.0–75.0)	50.0 (25.0–100.0)	50.0 (25.0–75.0)	
**Overall difficulty following dietary protein restriction**	n (missing)	33 (5)	38 (4)	23 (7)	0.4579
	Median (Q1–Q3)	0.0 (0.0–25.0)	0.0 (0.0–25.0)	0.0 (0.0–25.0)	

		Overall assessment of health status by the clinician[a]			
		**Good (n=38)**	**Very good (n=42)**	**Excellent (n=30)**	**p-value (Kruskal-Wallis)**

Phe: phenylalanine; PKU: phenylketonuria; Q: quartile; QOL: quality of life.

[a]Modality Poor (n=0) and Fair (n=0).

Table 3-2 - Clinical validity of the Adolescent PKU-QOL questionnaire: comparison of PKU-QOL scores according to severity of PKU defined by Phe levels at diagnosis in the adolescent evaluable population (n=110)

Scores of 0 indicate no impact (or no symptom for symptom scores), scores of 25 indicate a mild impact (or rare symptoms), scores of 50 indicate a moderate impact (or occasional symptoms), scores of 75 indicate major impact (or frequent symptom), scores of 100 indicate extremely severe impact (or very frequent symptoms)

		**Severity of PKU**		
		**Mild-moderate PKU (Phe level: 600-1200 µmol/L) (n=33)**	**Classic PKU (Phe level: >1200 µmol/L) (n=75)**	**p-value (Mann-Whitney /Wilcoxon)**
**Self-rated health status**	n (missing)	31 (2)	72 (3)	0.0117
	Median (Q1–Q3)	25.0 (0.0–50.0)	50.0 (25.0–50.0)	
**Headaches**	n (missing)	31 (2)	72 (3)	0.2448
	Median (Q1–Q3)	0.0 (0.0–25.0)	25.0 (0.0–37.5)	
**Stomach aches**	n (missing)	31 (2)	72 (3)	0.7159
	Median (Q1–Q3)	0.0 (0.0–25.0)	0.0 (0.0–25.0)	
**Tiredness**	n (missing)	31 (2)	72 (3)	0.6315
	Median (Q1–Q3)	50.0 (25.0–50.0)	50.0 (25.0–50.0)	
**Lack of concentration**	n (missing)	33 (0)	72 (3)	0.1403
	Median (Q1–Q3)	0.0 (0.0–25.0)	25.0 (0.0–50.0)	
**Slow thinking**	n (missing)	33 (0)	72 (3)	0.1714
	Median (Q1–Q3)	0.0 (0.0–25.0)	0.0 (0.0–25.0)	
**Irritability**	n (missing)	33 (0)	72 (3)	0.2132
	Median (Q1–Q3)	25.0 (0.0–25.0)	25.0 (0.0–50.0)	
**Aggressiveness**	n (missing)	33 (0)	72 (3)	0.4937
	Median (Q1–Q3)	0.0 (0.0–0.0)	0.0 (0.0–25.0)	
**Moodiness**	n (missing)	33 (0)	72 (3)	0.0302
	Median (Q1–Q3)	0.0 (0.0–25.0)	25.0 (0.0–50.0)	
**Sadness**	n (missing)	33 (0)	72 (3)	0.2927
	Median (Q1–Q3)	0.0 (0.0–0.0)	0.0 (0.0–25.0)	
**Anxiety**	n (missing)	33 (0)	72 (3)	1.0000
	Median (Q1–Q3)	0.0 (0.0–25.0)	0.0 (0.0–25.0)	
**Emotional impact of PKU**	n (missing)	32 (1)	72 (3)	0.1148
	Median (Q1–Q3)	27.5 (15.0–37.5)	30.0 (20.0–50.0)	
**Practical impact of PKU**	n (missing)	12 (21)	40 (35)	0.4404
	Median (Q1–Q3)	4.2 (0.0–16.7)	8.3 (0.0–25.0)	
**Social impact of PKU**	n (missing)	31 (2)	72 (3)	0.1055

		Severity of PKU		
		**Mild-moderate PKU (Phe level: 600-1200 µmol/L) (n=33)**	**Classic PKU (Phe level: >1200 µmol/L) (n=75)**	**p-value (Mann-Whitney /Wilcoxon)**
	Median (Q1–Q3)	8.3 (0.0–25.0)	16.7 (8.3–25.0)	
**Overall impact of PKU**	n (missing)	32 (1)	72 (3)	0.0674
	Median (Q1–Q3)	17.5 (11.3–25.0)	22.6 (13.1–34.5)	
**Anxiety – blood test**	n (missing)	32 (1)	72 (3)	0.6901
	Median (Q1–Q3)	0.0 (0.0–12.5)	0.0 (0.0–12.5)	
**Anxiety – Phe levels**	n (missing)	32 (1)	72 (3)	0.2554
	Median (Q1–Q3)	25.0 (0.0–50.0)	25.0 (12.5–62.5)	
**Adherence to supplements**	n (missing)	21 (12)	70 (5)	0.5896
	Median (Q1–Q3)	6.3 (0.0–16.7)	6.3 (0.0–25.0)	
**Practical impact of supplements**	n (missing)	21 (12)	72 (3)	0.0132
	Median (Q1–Q3)	6.3 (0.0–12.5)	18.8 (0.0–34.4)	
**Guilt if poor adherence to supplements**	n (missing)	22 (11)	71 (4)	0.3961
	Median (Q1–Q3)	25.0 (0.0–50.0)	25.0 (25.0–75.0)	
**Relationships within family because of supplements**	n (missing)	21 (12)	72 (3)	0.6050
	Median (Q1–Q3)	0.0 (0.0–25.0)	0.0 (0.0–25.0)	
**Taste – supplements**	n (missing)	21 (12)	72 (3)	0.0654
	Median (Q1–Q3)	50.0 (50.0–50.0)	37.5 (25.0–50.0)	
**Food temptations**	n (missing)	20 (13)	70 (5)	0.1055
	Median (Q1–Q3)	0.0 (0.0–37.5)	25.0 (0.0–50.0)	
**Adherence to dietary protein restriction**	n (missing)	20 (13)	68 (7)	0.1149
	Median (Q1–Q3)	0.0 (0.0–15.6)	10.4 (0.0–25.0)	
**Social impact of dietary protein restriction**	n (missing)	20 (13)	72 (3)	0.0642
	Median (Q1–Q3)	2.5 (0.0–5.0)	7.5 (0.0–25.0)	
**Practical impact of dietary protein restriction**	n (missing)	12 (21)	57 (18)	0.1878
	Median (Q1–Q3)	20.7 (13.7–28.6)	28.6 (15.0–35.0)	
**Overall impact of dietary protein restriction**	n (missing)	18 (15)	69 (6)	0.0176
	Median (Q1–Q3)	13.1 (3.1–18.2)	18.2 (11.4–27.8)	
**Taste – low-protein food**	n (missing)	14 (19)	69 (6)	0.6795
	Median (Q1–Q3)	25.0 (0.0–25.0)	25.0 (0.0–50.0)	
**Food enjoyment**	n (missing)	19 (14)	70 (5)	0.0477
	Median (Q1–Q3)	0.0 (0.0–0.0)	0.0 (0.0–25.0)	
**Guilt if dietary protein restriction not followed**	n (missing)	22 (11)	70 (5)	0.0217
	Median (Q1–Q3)	25.0 (0.0–50.0)	50.0 (25.0–75.0)	
**Overall difficulty following**	n (missing)	21 (12)	72 (3)	0.05[illegible]

		Severity of PKU		
		**Mild-moderate PKU (Phe level: 600-1200 µmol/L) (n=33)**	**Classic PKU (Phe level: >1200 µmol/L) (n=75)**	**p-value (Mann-Whitney /Wilcoxon)**
**dietary protein restriction**	Median (Q1–Q3)	0.0 (0.0–0.0)	0.0 (0.0–25.0)	

Phe: phenylalanine; PKU: phenylketonuria; Q: quartile; QOL: quality of life.
