# Supplementary material for: Development and psychometric validation of measures to assess the impact of phenylketonuria and its dietary treatment on patients’ and parents’ quality of life: the phenylketonuria – quality of life (PKU-QOL) questionnaires
Source: Orphanet J Rare Dis. 2015 May 10;10:59. doi: 10.1186/s13023-015-0261-6 (PMC4449597; doi:10.1186/s13023-015-0261-6)
Supplement: Additional file 4: — Clinical Validity of the Adult PKU-QOL. [file 13023_2015_261_MOESM4_ESM.pdf]

Additional file 04

Clinical validity of the Adult PKU-QOL questionnaire

Table 4-1 - Clinical validity of the Adult PKU-QOL questionnaire: comparison of PKU-QOL scores according to patient overall health status as rated by the clinician in the adult evaluable population (n=104)

Scores of 0 indicate no impact (or no symptom for symptom scores), scores of 25 indicate a mild impact (or rare symptoms), scores of 50 indicate a moderate impact (or occasional symptoms), scores of 75 indicate major impact (or frequent symptom), scores of 100 indicate extremely severe impact (or very frequent symptoms)

		Overall assessment of health status by the clinician[a]				
		**Fair (n=3)**	**Good (n=28)**	**Very good (n=46)**	**Excellent (n=26)**	**p-value (Kruskal-Wallis)**
**Self-rated health status**	n (missing)	3 (0)	27 (1)	46 (0)	26 (0)	0.0003
	Median (Q1–Q3)	50.0 (50.0–100.0)	50.0 (25.0–50.0)	25.0 (25.0–50.0)	25.0 (0.0–50.0)	
**Headaches**	n (missing)	3 (0)	26 (2)	46 (0)	26 (0)	0.3947
	Median (Q1–Q3)	50.0 (0.0–50.0)	25.0 (0.0–50.0)	0.0 (0.0–25.0)	12.5 (0.0–25.0)	
**Stomach aches**	n (missing)	3 (0)	26 (2)	46 (0)	26 (0)	0.0126
	Median (Q1–Q3)	50.0 (25.0–75.0)	12.5 (0.0–50.0)	0.0 (0.0–25.0)	0.0 (0.0–0.0)	
**Tiredness**	n (missing)	3 (0)	26 (2)	46 (0)	26 (0)	0.4790
	Median (Q1–Q3)	50.0 (25.0–50.0)	50.0 (25.0–75.0)	50.0 (25.0–50.0)	37.5 (25.0–50.0)	
**Lack of concentration**	n (missing)	3 (0)	26 (2)	46 (0)	26 (0)	0.1596
	Median (Q1–Q3)	25.0 (25.0–50.0)	25.0 (0.0–50.0)	25.0 (0.0–50.0)	0.0 (0.0–25.0)	
**Slow thinking**	n (missing)	3 (0)	26 (2)	46 (0)	26 (0)	0.0377
	Median (Q1–Q3)	25.0 (0.0–50.0)	25.0 (0.0–50.0)	0.0 (0.0–25.0)	0.0 (0.0–0.0)	
**Trembling hands**	n (missing)	3 (0)	26 (2)	46 (0)	26 (0)	0.5172
	Median (Q1–Q3)	0.0 (0.0–50.0)	0.0 (0.0–25.0)	0.0 (0.0–0.0)	0.0 (0.0–25.0)	
**Irritability**	n (missing)	3 (0)	27 (1)	46 (0)	26 (0)	0.0499
	Median (Q1–Q3)	75.0 (50.0–75.0)	50.0 (0.0–50.0)	50.0 (25.0–50.0)	25.0 (0.0–50.0)	
**Aggressiveness**	n (missing)	3 (0)	26 (2)	46 (0)	26 (0)	0.2428
	Median (Q1–Q3)	50.0 (0.0–75.0)	0.0 (0.0–25.0)	0.0 (0.0–25.0)	0.0 (0.0–25.0)	
**Moodiness**	n (missing)	3 (0)	26 (2)	46 (0)	26 (0)	0.2972
	Median (Q1–Q3)	50.0 (0.0–50.0)	25.0 (0.0–50.0)	25.0 (0.0–50.0)	0.0 (0.0–25.0)	
**Sadness**	n (missing)	3 (0)	26 (2)	46 (0)	26 (0)	0.0437
	Median (Q1–Q3)	50.0 (25.0–75.0)	50.0 (0.0–75.0)	25.0 (0.0–50.0)	0.0 (0.0–50.0)	
**Anxiety**	n (missing)	3 (0)	26 (2)	46 (0)	26 (0)	0.1295
	Median (Q1–Q3)	75.0 (0.0–75.0)	12.5 (0.0–50.0)	0.0 (0.0–25.0)	25.0 (0.0–50.0)	

		Overall assessment of health status by the clinician[a]				
		**Fair (n=3)**	**Good (n=28)**	**Very good (n=46)**	**Excellent (n=26)**	**p-value (Kruskal-Wallis)**
**Emotional impact of PKU**	n (missing)	3 (0)	27 (1)	46 (0)	26 (0)	<0.0001
	Median (Q1–Q3)	55.0 (45.0–65.0)	60.0 (45.0–70.0)	45.0 (25.0–55.0)	30.0 (20.0–40.0)	
**Practical impact of PKU**	n (missing)	3 (0)	26 (2)	43 (3)	26 (0)	0.0231
	Median (Q1–Q3)	18.8 (8.3–37.5)	25.0 (6.3–37.5)	16.7 (8.3–25.0)	6.3 (0.0–12.5)	
**Social impact of PKU**	n (missing)	3 (0)	27 (1)	46 (0)	26 (0)	0.1726
	Median (Q1–Q3)	12.5 (8.3–62.5)	18.8 (6.3–37.5)	14.6 (6.3–25.0)	10.4 (6.3–18.8)	
**Overall impact of PKU**	n (missing)	2 (1)	17 (11)	26 (20)	14 (12)	0.0039
	Median (Q1–Q3)	41.7 (31.3–52.1)	39.6 (29.2–45.8)	27.1 (18.8–37.5)	17.7 (12.5–27.1)	
**Anxiety – blood test**	n (missing)	2 (1)	23 (5)	44 (2)	24 (2)	0.2178
	Median (Q1–Q3)	18.8 (12.5–25.0)	0.0 (0.0–0.0)	0.0 (0.0–12.5)	0.0 (0.0–12.5)	
**Anxiety – Phe levels**	n (missing)	3 (0)	27 (1)	46 (0)	26 (0)	0.1703
	Median (Q1–Q3)	75.0 (50.0–100.0)	25.0 (0.0–50.0)	25.0 (25.0–50.0)	25.0 (25.0–75.0)	
**Anxiety – Phe levels during pregnancy**	n (missing)	3 (0)	13 (15)	32 (14)	16 (10)	0.0921
	Median (Q1–Q3)	25.0 (0.0–75.0)	100.0 (75.0–100.0)	100.0 (75.0–100.0)	100.0 (50.0–100.0)	
**Financial impact of PKU**	n (missing)	3 (0)	27 (1)	46 (0)	26 (0)	0.0350
	Median (Q1–Q3)	25.0 (0.0–100.0)	25.0 (0.0–50.0)	0.0 (0.0–25.0)	0.0 (0.0–25.0)	
**Information on PKU**	n (missing)	3 (0)	27 (1)	46 (0)	26 (0)	0.2653
	Median (Q1–Q3)	50.0 (0.0–100.0)	50.0 (25.0–50.0)	25.0 (25.0–50.0)	25.0 (25.0–50.0)	
**Adherence to supplements**	n (missing)	2 (1)	19 (9)	32 (14)	18 (8)	0.9908
	Median (Q1–Q3)	16.7 (8.3–25.0)	16.7 (8.3–33.3)	16.7 (4.2–33.3)	8.3 (0.0–41.7)	
**Practical impact of supplements**	n (missing)	3 (0)	21 (7)	37 (9)	20 (6)	0.0327
	Median (Q1–Q3)	68.8 (37.5–75.0)	18.8 (6.3–37.5)	18.8 (0.0–37.5)	12.5 (0.0–31.3)	
**Guilt if poor adherence to supplements**	n (missing)	3 (0)	23 (5)	37 (9)	18 (8)	0.9693
	Median (Q1–Q3)	25.0 (0.0–100.0)	50.0 (25.0–75.0)	50.0 (25.0–75.0)	50.0 (25.0–75.0)	
**Relationships within family because of supplements**	n (missing)	3 (0)	22 (6)	37 (9)	20 (6)	0.6335
	Median (Q1–Q3)	0.0 (0.0–25.0)	0.0 (0.0–0.0)	0.0 (0.0–25.0)	0.0 (0.0–25.0)	
**Taste – supplements**	n (missing)	3 (0)	21 (7)	37 (9)	19 (7)	0.5430
	Median (Q1–Q3)	50.0 (50.0–75.0)(	50.0 (25.0–75.0)	50.0 (25.0–50.0)	50.0 (25.0–75.0)	
**Food temptations**	n (missing)	3 (0)	24 (4)	38 (8)	19 (7)	0.0377
	Median (Q1–Q3)	50.0 (37.5–62.5)	50.0 (25.0–62.5)	43.8 (12.5–50.0)	25.0 (0.0–50.0)	
**Adherence to dietary protein restriction**	n (missing)	2 (1)	22 (6)	36 (10)	19 (7)	0.6712
	Median (Q1–Q3)	12.5 (0.0-25.0)	18.8 (6.3-30.0)	22.5 (0.0-35.0)	10.0 (0.0-30.0)	
**Social impact of dietary protein restriction**	n (missing)	3 (0)	23 (5)	28 (8)	20 (6)	0.0643
	Median (Q1–Q3)	58.3 (16.7–79.2)	16.7 (8.3–35.0)	11.3 (4.2–20.8)	8.3 (0.0–20.8)	

		Overall assessment of health status by the clinician[a]				
		**Fair (n=3)**	**Good (n=28)**	**Very good (n=46)**	**Excellent (n=26)**	**p-value (Kruskal-Wallis)**
**Practical impact of dietary protein restriction**	n (missing)	3 (0)	22 (6)	32 (14)	15 (11)	0.0789
	Median (Q1–Q3)	71.4 (46.4–75.0)	39.6 (21.4–54.2)	30.4 (21.4–48.2)	32.1 (12.5–57.1)	
**Overall impact of dietary protein restriction**	n (missing)	3 (0)	22 (6)	34 (12)	17 (9)	0.0631
	Median (Q1–Q3)	67.3 (32.7–75.0)	27.1 (18.8–40.0)	22.8 (11.5–35.4)	20.8 (6.8–32.7)	
**Taste – low-protein food**	n (missing)	3 (0)	19 (9)	32 (14)	14 (12)	0.1245
	Median (Q1–Q3)	50.0 (50.0–75.0)	25.0 (25.0–50.0)	25.0 (25.0–37.5)	25.0 (25.0–50.0)	
**Food enjoyment**	n (missing)	3 (0)	23 (5)	38 (8)	19 (7)	0.3347
	Median (Q1–Q3)	75.0 (0.0–100.0)	25.0 (0.0–50.0)	25.0 (0.0–50.0)	25.0 (0.0–50.0)	
**Guilt if dietary protein restriction not followed**	n (missing)	3 (0)	22 (6)	38 (8)	20 (6)	0.8542
	Median (Q1–Q3)	25.0 (25.0–100.0)	50.0 (25.0–75.0)	50.0 (25.0–100.0)	50.0 (25.0–75.0)	
**Overall difficulty following dietary protein restriction**	n (missing)	3 (0)	23 (5)	39 (7)	19 (7)	0.3826
	Median (Q1–Q3)	50.0 (0.0–75.0)	25.0 (0.0–50.0)	25.0 (0.0–50.0)	25.0 (0.0–50.0)	

Phe: phenylalanine; PKU: phenylketonuria; Q: quartile; QOL: quality of life.

[a]Modality Poor (n=0).

Table 4-2 - Clinical validity of the Adult PKU-QOL questionnaire: comparison of PKU-QoL scores according to severity of PKU defined by Phe levels at diagnosis in the adult evaluable population (n=104)

Scores of 0 indicate no impact (or no symptom for symptom scores), scores of 25 indicate a mild impact (or rare symptoms), scores of 50 indicate a moderate impact (or occasional symptoms), scores of 75 indicate major impact (or frequent symptom), scores of 100 indicate extremely severe impact (or very frequent symptoms)

		Severity of PKU		
		**Mild-moderate PKU (Phe level: 600-1200 µmol/L) (n=37)**	**Classic PKU (Phe level: >1200 µmol/L) (n=67)**	**p-value (Mann-Whitney /Wilcoxon)**
**Self-rated health status**	n (missing)	36 (1)	67 (0)	0.3698
	Median (Q1–Q3)	50.0 (25.0–50.0)	25.0 (25.0–50.0)	
**Headaches**	n (missing)	36 (1)	66 (1)	0.4257
	Median (Q1–Q3)	25.0 (0.0–50.0)	12.5 (0.0–25.0)	
**Stomach aches**	n (missing)	36 (1)	66 (1)	0.3965
	Median (Q1–Q3)	0.0 (0.0–37.5)	0.0 (0.0–25.0)	
**Tiredness**	n (missing)	36 (1)	66 (1)	0.9446
	Median (Q1–Q3)	50.0 (25.0–50.0)	50.0 (25.0–50.0)	
**Lack of concentration**	n (missing)	36 (1)	66 (1)	0.8232
	Median (Q1–Q3)	25.0 (0.0–50.0)	25.0 (0.0–50.0)	
**Slow thinking**	n (missing)	36 (1)	66 (1)	0.2678
	Median (Q1–Q3)	0.0 (0.0–25.0)	0.0 (0.0–25.0)	
**Trembling hands**	n (missing)	36 (1)	66 (1)	0.9893
	Median (Q1–Q3)	0.0 (0.0–25.0)	0.0 (0.0–25.0)	
**Irritability**	n (missing)	36 (1)	67 (0)	0.7517
	Median (Q1–Q3)	37.5 (0.0–50.0)	25.0 (25.0–50.0)	
**Aggressiveness**	n (missing)	36 (1)	66 (1)	0.6881
	Median (Q1–Q3)	0.0 (0.0–25.0)	0.0 (0.0–25.0)	
**Moodiness**	n (missing)	36 (1)	66 (1)	0.9192
	Median (Q1–Q3)	25.0 (0.0–50.0)	25.0 (0.0–50.0)	
**Sadness**	n (missing)	36 (1)	66 (1)	0.7331
	Median (Q1–Q3)	25.0 (0.0–50.0)	25.0 (0.0–50.0)	
**Anxiety**	n (missing)	36 (1)	66 (1)	0.1801
	Median (Q1–Q3)	25.0 (0.0–50.0)	0.0 (0.0–25.0)	
**Emotional impact of PKU**	n (missing)	36 (1)	67 (0)	0.5859
	Median (Q1–Q3)	45.0 (25.0–60.0)	45.0 (30.0–65.0)	

		Severity of PKU		
		**Mild-moderate PKU (Phe level: 600-1200 µmol/L) (n=37)**	**Classic PKU (Phe level: >1200 µmol/L) (n=67)**	**p-value (Mann-Whitney /Wilcoxon)**
**Practical impact of PKU**	n (missing)	33 (4)	66 (1)	0.3205
	Median (Q1–Q3)	8.3 (0.0–25.0)	16.7 (6.3–31.3)	
**Social impact of PKU**	n (missing)	36 (1)	67 (0)	0.2991
	Median (Q1–Q3)	12.5 (6.3–18.8)	16.7 (6.3 – 25.0)	
**Overall impact of PKU**	n (missing)	16 (21)	44 (23)	0.3793
	Median (Q1–Q3)	25.0 (17.7–32.3)	28.1 (18.8–41.7)	
**Anxiety – blood test**	n (missing)	33 (4)	61 (6)	0.2108
	Median (Q1–Q3)	0.0 (0.0–25.0)	0.0 (0.0–12.5)	
**Anxiety – Phe levels**	n (missing)	36 (1)	67 (0)	0.9142
	Median (Q1–Q3)	25.0 (25.0–50.0)	25.0 (25.0–50.0)	
**Anxiety – Phe levels during pregnancy**	n (missing)	23 (14)	42 (25)	0.2571
	Median (Q1–Q3)	75.0 (50.0–100.0)	100.0 (75.0–100.0)	
**Financial impact of PKU**	n (missing)	36 (1)	67 (0)	0.0174
	Median (Q1–Q3)	0.0 (0.0–25.0)	25.0 (0.0–50.0)	
**Information on PKU**	n (missing)	36 (1)	67 (0)	0.7770
	Median (Q1–Q3)	25.0 (25.0–50.0)	25.0 (25.0–50.0)	
**Adherence to supplements**	n (missing)	17 (20)	55 (12)	0.6323
	Median (Q1–Q3)	8.3 (0.0–25.0)	16.7 (0.0–33.3)	
**Practical impact of supplements**	n (missing)	19 (18)	63 (4)	0.8370
	Median (Q1–Q3)	12.5 (6.3–31.3)	18.8 (0.0–37.5)	
**Guilt if poor adherence to supplements**	n (missing)	19 (18)	63 (4)	0.9776
	Median (Q1–Q3)	50.0 (25.0–75.0)	50.0 (25.0–75.0)	
**Relationships within family because of supplements**	n (missing)	19 (18)	64 (3)	0.2339
	Median (Q1–Q3)	0.0 (0.0–0.0)	0.0 (0.0–25.0)	
**Taste – supplements**	n (missing)	19 (18)	62 (5)	0.8918
	Median (Q1–Q3)	50.0 (25.0–50.0)	50.0 (25.0–75.0)	
**Food temptations**	n (missing)	24 (13)	61 (6)	0.5256
	Median (Q1–Q3)	37.5 (25.0–50.0)	50.0 (12.5–50.0)	
**Adherence to dietary protein restriction**	n (missing)	21 (16)	59 (8)	0.1498
	Median (Q1–Q3)	15.0 (0.0–25.0)	20.0 (0.0–37.5)	
**Social impact of dietary protein restriction**	n (missing)	24 (13)	61 (6)	0.3600
	Median (Q1–Q3)	9.2 (0.0–29.6)	12.5 (8.3–20.8)	

		Severity of PKU		
		**Mild-moderate PKU (Phe level: 600-1200 µmol/L) (n=37)**	**Classic PKU (Phe level: >1200 µmol/L) (n=67)**	**p-value (Mann-Whitney /Wilcoxon)**
**Practical impact of dietary protein restriction**	n (missing)	21 (16)	52 (15)	0.9708
	Median (Q1–Q3)	32.1 (20.8–46.4)	35.7 (19.6–51.8)	
**Overall impact of dietary protein restriction**	n (missing)	22 (15)	55 (12)	0.9865
	Median (Q1–Q3)	26.1 (11.5–34.1)	25.0 (11.4–38.5)	
**Taste – low-protein food**	n (missing)	16 (21)	53 (14)	0.4093
	Median (Q1–Q3)	25.0 (25.0–50.0)	25.0 (25.0–50.0)	
**Food enjoyment**	n (missing)	24 (13)	60 (7)	0.4831
	Median (Q1–Q3)	25.0 (0.0–25.0)	25.0 (0.0–50.0)	
**Guilt if dietary protein restriction not followed**	n (missing)	23 (14)	61 (6)	0.7968
	Median (Q1–Q3)	50.0 (25.0–75.0)	50.0 (25.0–75.0)	
**Overall difficulty following dietary protein restriction**	n (missing)	24 (13)	61 (6)	0.7708
	Median (Q1–Q3)	25.0 (0.0–50.0)	25.0 (0.0–50.0)	

Phe: phenylalanine; PKU: phenylketonuria; Q: quartile; QOL: quality of life.
