# Supplementary material for: Development and psychometric validation of measures to assess the impact of phenylketonuria and its dietary treatment on patients’ and parents’ quality of life: the phenylketonuria – quality of life (PKU-QOL) questionnaires
Source: Orphanet J Rare Dis. 2015 May 10;10:59. doi: 10.1186/s13023-015-0261-6 (PMC4449597; doi:10.1186/s13023-015-0261-6)
Supplement: Additional file 5: — Clinical Validity of the Parent PKU-QOL. [file 13023_2015_261_MOESM5_ESM.pdf]

Additional file 05

Clinical validity of the Parent PKU-QoL questionnaire

Table 5-1 - Clinical validity of the Parent PKU-QOL Questionnaire: comparison of PKU-QOL scores according to patient overall health status as rated by the clinician in the parent evaluable population (n=253)

Scores of 0 indicate no impact (or no symptom for symptom scores), scores of 25 indicate a mild impact (or rare symptoms), scores of 50 indicate a moderate impact (or occasional symptoms), scores of 75 indicate major impact (or frequent symptom), scores of 100 indicate extremely severe impact (or very frequent symptoms)

		Overall health status as rated by the clinician[a]				
		**Fair (n=2)**	**Good (n=64)**	**Very good (n=101)**	**Excellent (n=84)**	**p-value (Kruskal-Wallis)**
**Child health status**	n (missing)	2 (0)	61 (3)	99 (2)	84 (0)	0.0002
	Median (Q1–Q3)	12.5 (0.0–25.0)	50.0 (25.0–50.0)	25.0 (25.0–50.0)	25.0 (0.0–37.5)	
**Headaches**	n (missing)	2 (0)	61 (3)	99 (2)	84 (0)	0.0119
	Median (Q1–Q3)	25.0 (0.0–50.0)	25.0 (0.0–25.0)	0.0 (0.0–25.0)	0.0 (0.0–12.5)	
**Stomach aches**	n (missing)	2 (0)	61 (3)	99 (2)	84 (0)	0.3913
	Median (Q1–Q3)	37.5 (0.0–75.0)	0.0 (0.0–25.0)	25.0 (0.0–50.0)	0.0 (0.0–25.0)	
**Tiredness**	n (missing)	2 (0)	61 (3)	99 (2)	84 (0)	0.3131
	Median (Q1–Q3)	12.5 (0.0–25.0)	50.0 (25.0–50.0)	25.0 (0.0–50.0)	25.0 (0.0–50.0)	
**Lack of concentration**	n (missing)	2 (0)	60 (4)	97 (4)	82 (2)	0.2777
	Median (Q1–Q3)	12.5 (0.0–25.0)	25.0 (0.0–50.0)	25.0 (0.0–50.0)	25.0 (0.0–50.0)	
**Slow thinking**	n (missing)	2 (0)	58 (6)	96 (5)	80 (4)	0.0533
	Median (Q1–Q3)	12.5 (0.0–25.0)	25.0 (0.0–50.0)	0.0 (0.0–25.0)	0.0 (0.0–25.0)	
**Irritability**	n (missing)	2 (0)	61 (3)	99 (2)	84 (0)	0.0150
	Median (Q1–Q3)	62.5 (50.0–75.0)	50.0 (25.0–75.0)	25.0 (25.0–50.0)	25.0 (0.0–50.0)	
**Aggressiveness**	n (missing)	2 (0)	59 (5)	98 (3)	82 (2)	0.0853
	Median (Q1–Q3)	37.5 (25.0–50.0)	0.0 (0.0–25.0)	0.0 (0.0–25.0)	0.0 (0.0–25.0)	
**Moodiness**	n (missing)	2 (0)	61 (3)	98 (3)	83 (1)	0.2911
	Median (Q1–Q3)	12.5 (0.0–25.0)	25.0 (25.0–50.0)	25.0 (0.0–50.0)	25.0 (0.0–50.0)	
**Sadness**	n (missing)	2 (0)	61 (3)	99 (2)	82 (2)	0.0013
	Median (Q1–Q3)	25.0 (25.0–25.0)	25.0 (0.0–50.0)	25.0 (0.0–25.0)	0.0 (0.0–25.0)	
**Anxiety**	n (missing)	2 (0)	59 (5)	97 (4)	83 (1)	0.0658
	Median (Q1–Q3)	0.0 (0.0–0.0)	0.0 (0.0–25.0)	0.0 (0.0–25.0)	0.0 (0.0–25.0)	
**Emotional impact of PKU**	n (missing)	2 (0)	61 (3)	98 (3)	84 (0)	0.0007
	Median (Q1–Q3)	28.1 (18.8–37.5)	56.3 (31.3–75.0)	37.5 (25.0–56.3)	34.4 (18.8–53.1)	
**Practical impact**	n (missing)	2 (0)	45 (19)	73 (28)	69 (15)	0.0066

		Overall health status as rated by the clinician[a]				
		**Fair (n=2)**	**Good (n=64)**	**Very good (n=101)**	**Excellent (n=84)**	**p-value (Kruskal-Wallis)**
**of PKU**	Median (Q1–Q3)	25.4 (5.0–45.8)	16.7 (4.2–29.2)	12.5 (4.2–20.8)	5.0 (0.0–12.5)	
**Social impact of PKU**	n (missing)	2 (0)	59 (5)	95 (6)	83 (1)	0.0189
	Median (Q1–Q3)	25.0 (10.0–40.0)	15.0 (10.0–31.3)	10.0 (5.0–25.0)	10.0 (5.0–20.0)	
**Overall impact of PKU**	n (missing)	2 (0)	61 (3)	97 (4)	84 (0)	0.0003
	Median (Q1–Q3)	26.2 (10.7–41.7)	28.3 (17.9–41.7)	21.2 (10.7–33.3)	15.8 (8.6–26.7)	
**Anxiety – blood test**	n (missing)	2 (0)	61 (3)	97 (4)	82 (2)	0.6697
	Median (Q1–Q3)	25.0 (25.0–25.0)	12.5 (0.0–37.5)	12.5 (0.0–25.0)	12.5 (0.0–37.5)	
**Impact of child anxiety – blood test**	n (missing)	2 (0)	59 (5)	97 (4)	82 (2)	0.7835
	Median (Q1–Q3)	25.0 (12.5–37.5)	25.0 (0.0–50.0)	12.5 (0.0–37.5)	12.5 (0.0–37.5)	
**Anxiety – Phe levels**	n (missing)	2 (0)	61 (3)	98 (3)	84 (0)	0.2060
	Median (Q1–Q3)	50.0 (25.0–75.0)	75.0 (50.0–75.0)	50.0 (25.0–75.0)	50.0 (25.0–75.0)	
**Financial impact of PKU**	n (missing)	2 (0)	60 (4)	97 (4)	84 (0)	0.0039
	Median (Q1–Q3)	75.0 (50.0–100.0)	25.0 (0.0–50.0)	25.0 (0.0–50.0)	0.0 (0.0–25.0)	
**Information on PKU**	n (missing)	2 (0)	61 (3)	98 (3)	84 (0)	0.0321
	Median (Q1–Q3)	37.5 (0.0–75.0)	25.0 (25.0–50.0)	25.0 (25.0–50.0)	25.0 (0.0–37.5)	
**Adherence to supplements**	n (missing)	2 (0)	55 (9)	88 (13)	70 (14)	0.2241
	Median (Q1–Q3)	0.0 (0.0–0.0)	0.0 (0.0–25.0)	0.0 (0.0–25.0)	0.0 (0.0–25.0)	
**Management of supplements**	n (missing)	2 (0)	55 (9)	89 (12)	71 (13)	0.1802
	Median (Q1–Q3)	37.5 (25.0–50.0)	0.0 (0.0–25.0)	0.0 (0.0–50.0)	0.0 (0.0–25.0)	
**Practical impact of supplements**	n (missing)	2 (0)	55 (9)	89 (12)	69 (15)	0.3529
	Median (Q1–Q3)	25.0 (16.7–33.3)	8.3 (0.0–33.3)	16.7 (0.0–33.3)	8.3 (0.0–33.3)	
**Guilt if poor adherence to supplements**	n (missing)	2 (0)	52 (12)	89 (12)	69 (15)	0.6966
	Median (Q1–Q3)	62.5 (25.0–100.0)	50.0 (25.0–100.0)	50.0 (25.0–75.0)	50.0 (25.0–75.0)	
**Relationships within family because of supplements**	n (missing)	2 (0)	56 (8)	89 (12)	68 (16)	0.2459
	Median (Q1–Q3)	12.5 (0.0–25.0)	0.0 (0.0–25.0)	25.0 (0.0–50.0)	0.0 (0.0–25.0)	
**Adherence to dietary protein restriction**	n (missing)	2 (0)	55 (9)	91 (10)	70 (14)	0.0011
	Median (Q1–Q3)	12.5 (0.0–25.0)	0.0 (0.0–25.0)	0.0 (0.0–25.0)	0.0 (0.0–0.0)	
**Management of dietary protein restriction**	n (missing)	2 (0)	56 (8)	92 (9)	70 (14)	0.0484
	Median (Q1–Q3)	37.5 (25.0–50.0)	25.0 (12.5–47.9)	16.7 (4.2–37.5)	16.7 (4.2–33.3)	
**Practical impact of dietary protein restriction**	n (missing)	2 (0)	55 (9)	88 (13)	63 (21)	0.8604
	Median (Q1–Q3)	34.5 (33.3–35.7)	32.1 (17.9–50.0)	30.4 (17.3–46.4)	28.6 (10.7–46.4)	
**Food enjoyment**	n (missing)	2 (0)	56 (8)	91 (10)	67 (17)	0.8996
	Median (Q1–Q3)	12.5 (0.0–25.0)	25.0 (0.0–25.0)	0.0 (0.0–25.0)	25.0 (0.0–25.0)	

		Overall health status as rated by the clinician[a]				
		**Fair (n=2)**	**Good (n=64)**	**Very good (n=101)**	**Excellent (n=84)**	**p-value (Kruskal-Wallis)**
**Guilt if dietary protein restriction not followed**	n (missing)	2 (0)	53 (11)	92 (9)	71 (13)	0.7164
	Median (Q1–Q3)	50.0 (25.0–75.0)	50.0 (25.0–75.0)	50.0 (25.0–75.0)	25.0 (0.0 – 75.0)	

Phe: phenylalanine; PKU: phenylketonuria; Q: quartile; QOL: quality of life.

[a]Modality Poor (n=0).

Table 5-2 - Clinical validity of the Parent PKU-QOL Questionnaire: comparison of PKU-QOL scores according to severity of PKU defined by Phe levels at diagnosis in the parent evaluable population (n=253)

Scores of 0 indicate no impact (or no symptom for symptom scores), scores of 25 indicate a mild impact (or rare symptoms), scores of 50 indicate a moderate impact (or occasional symptoms), scores of 75 indicate major impact (or frequent symptom), scores of 100 indicate extremely severe impact (or very frequent symptoms)

		Severity of PKU		p-value (Mann-Whitney /Wilcoxon)
		Mild-moderate PKU (Phe level: 600-1200 µmol/L) (n=75)	Classic PKU (Phe level: >1200 µmol/L) (n=172)	
**Parent-rated health status**	n (missing)	75 (0)	167 (5)	0.0095
	Median (Q1–Q3)	25.0 (0.0–50.0)	25.0 (25.0–50.0)	
**Headaches**	n (missing)	75 (0)	167 (5)	0.2009
	Median (Q1–Q3)	0.0 (0.0–25.0)	0.0 (0.0–25.0)	
**Stomach aches**	n (missing)	75 (0)	167 (5)	0.4817
	Median (Q1–Q3)	0.0 (0.0–50.0)	0.0 (0.0–25.0)	
**Tiredness**	n (missing)	75 (0)	167 (5)	0.7532
	Median (Q1–Q3)	25.0 (0.0–50.0)	25.0 (0.0–50.0)	
**Lack of concentration**	n (missing)	74 (1)	163 (9)	0.6341
	Median (Q1–Q3)	25.0 (0.0–50.0)	25.0 (0.0–50.0)	
**Slow thinking**	n (missing)	73 (2)	159 (13)	0.3372
	Median (Q1–Q3)	0.0 (0.0–25.0)	0.0 (0.0–50.0)	
**Irritability**	n (missing)	75 (0)	167 (5)	0.0531
	Median (Q1–Q3)	25.0 (0.0–50.0)	50.0 (25.0–50.0)	
**Aggressiveness**	n (missing)	73 (2)	164 (8)	0.6625
	Median (Q1–Q3)	0.0 (0.0–25.0)	0.0 (0.0–25.0)	
**Moodiness**	n (missing)	74 (1)	166 (6)	0.9882
	Median (Q1–Q3)	25.0 (0.0–50.0)	25.0 (0.0–50.0)	
**Sadness**	n (missing)	73 (2)	167 (5)	0.2872
	Median (Q1–Q3)	0.0 (0.0–25.0)	25.0 (0.0–25.0)	
**Anxiety**	n (missing)	73 (2)	164 (8)	0.6344
	Median (Q1–Q3)	0.0 (0.0–25.0)	0.0 (0.0–25.0)	
**Emotional impact of PKU**	n (missing)	74 (1)	167 (5)	0.0231
	Median (Q1–Q3)	34.4 (18.8–56.3)	43.8 (25.0–62.5)	
**Practical impact of PKU**	n (missing)	59 (16)	126 (46)	0.0034
	Median (Q1–Q3)	5.0 (0.0–16.7)	12.5 (4.2–25.0)	

		Severity of PKU		p-value (Mann-Whitney /Wilcoxon)
		Mild-moderate PKU (Phe level: 600-1200 µmol/L) (n=75)	Classic PKU (Phe level: >1200 µmol/L) (n=172)	
**Social impact of PKU**	n (missing)	72 (3)	163 (9)	0.1628
	Median (Q1–Q3)	10.0 (5.0–20.0)	15.0 (5.0–25.0)	
**Overall impact of PKU**	n (missing)	73 (2)	167 (5)	0.0035
	Median (Q1–Q3)	18.3 (8.3–26.7)	23.2 (12.5–36.5)	
**Anxiety – blood test**	n (missing)	73 (2)	165 (7)	0.1435
	Median (Q1–Q3)	12.5 (0.0–25.0)	12.5 (0.0–37.5)	
**Impact of child anxiety – blood test**	n (missing)	73 (2)	163 (9)	0.4306
	Median (Q1–Q3)	12.5 (0.0–37.5)	12.5 (0.0–37.5)	
**Anxiety – Phe levels**	n (missing)	74 (1)	167 (5)	0.3518
	Median (Q1–Q3)	50.0 (25.0–75.0)	50.0 (25.0–75.0)	
**Financial impact of PKU**	n (missing)	73 (2)	166 (6)	0.0028
	Median (Q1–Q3)	0.0 (0.0–25.0)	25.0 (0.0–50.0)	
**Information on PKU**	n (missing)	74 (1)	167 (5)	0.3909
	Median (Q1–Q3)	25.0 (25.0–50.0)	25.0 (25.0–50.0)	
**Adherence to supplements**	n (missing)	49 (26)	163 (9)	0.8713
	Median (Q1–Q3)	0.0 (0.0–25.0)	0.0 (0.0–25.0)	
**Management of supplements**	n (missing)	49 (26)	165 (7)	0.6052
	Median (Q1–Q3)	0.0 (0.0–25.0)	0.0 (0.0–25.0)	
**Practical impact of supplements**	n (missing)	49 (26)	163 (9)	0.0409
	Median (Q1–Q3)	8.3 (0.0–16.7)	16.7 (0.0–41.7)	
**Guilt if poor adherence to supplements**	n (missing)	50 (25)	159 (13)	0.8627
	Median (Q1–Q3)	50.0 (25.0–75.0)	50.0 (25.0–75.0)	
**Relationships within family because of supplements**	n (missing)	49 (26)	163 (9)	0.7388
	Median (Q1–Q3)	0.0 (0.0–25.0)	0.0 (0.0–25.0)	
**Adherence to dietary protein restriction**	n (missing)	52 (23)	163 (9)	0.0982
	Median (Q1–Q3)	0.0 (0.0–0.0)	0.0 (0.0–25.0)	
**Management of dietary protein restriction**	n (missing)	52 (23)	165 (7)	0.0987
	Median (Q1–Q3)	16.7 (4.2–29.2)	20.8 (8.3–41.7)	
**Practical impact of dietary protein restriction**	n (missing)	43 (32)	162 (10)	0.0695
	Median (Q1–Q3)	28.6 (10.7–37.5)	32.7 (16.7–50.0)	
**Food enjoyment**	n (missing)	49 (26)	164 (8)	0.0032
	Median (Q1–Q3)	0.0 (0.0–25.0)	25.0 (0.0–37.5)	
**Guilt if dietary protein restriction not followed**	n (missing)	54 (21)	161 (11)	0.4313
	Median (Q1–Q3)	25.0 (25.0–50.0)	50.0 (25.0–75.0)	

Phe: phenylalanine; PKU: phenylketonuria; Q: quartile; QOL: quality of life.
